# Nano Delivery System for Atherosclerosis

**DOI:** 10.3390/jfb16010002

**Published:** 2024-12-24

**Authors:** Zhuoyi Rong, Xuan He, Tianjian Fan, Haitao Zhang

**Affiliations:** Hunan Province Cooperative Innovation Center for Molecular Target New Drug Study, School of Pharmaceutical Science, Hengyang Medical School, University of South China, Hengyang 421001, China

**Keywords:** nanoparticle, nanomaterial, theranostics, atherosclerosis, atherosclerotic plaque

## Abstract

Atherosclerosis, a pathological process propelled by inflammatory mediators and lipids, is a principal contributor to cardiovascular disease incidents. Currently, drug therapy, the primary therapeutic strategy for atherosclerosis, faces challenges such as poor stability and significant side effects. The advent of nanomaterials has garnered considerable attention from scientific researchers. Nanoparticles, such as liposomes and polymeric nanoparticles, have been developed for drug delivery in atherosclerosis treatment. This review will focus on how nanoparticles effectively improve drug safety and efficacy, as well as the continuous development and optimization of nanoparticles of the same material and further explore current challenges and future opportunities in this field.

## 1. Introduction

**Figure 1:**
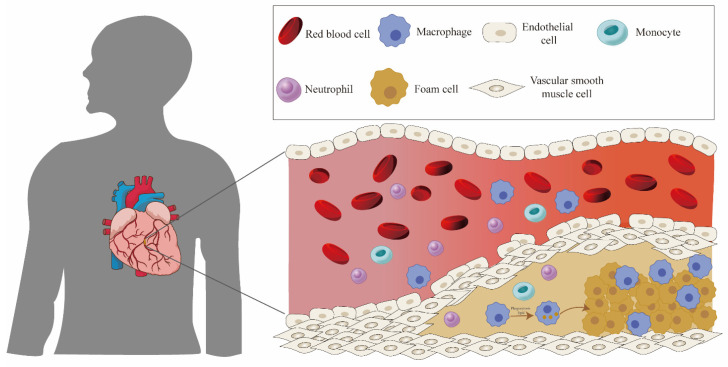
The process of foam cell formation. The accumulation of lipids at blood vessels destroys vascular endothelial cells, causing them to release inflammatory factors that induce the body’s immune cells (macrophages, monocytes, and neutrophils) to accumulate toward them. Macrophages reach the lipid accumulation and phagocytose large amounts of lipids, forming foam cells.

Cardiovascular diseases (CVDs) are the leading cause of non-communicable disease-related deaths, accounting for approximately 17.9 million lives lost annually [1,2]. The primary cause of many cardiovascular disorders is believed to be atherosclerosis (AS), an inflammatory and lipid-driven pathological condition that significantly contributes to the incidence of cardiovascular diseases [3]. Initially, substantial lipid accumulation occurs on the arterial walls, which is then oxidized by reactive oxygen species (ROS) to form low-density lipoproteins (ox-LDL) [4]. Ox-LDL causes damage to endothelial cells, triggering a cascade of inflammatory responses. Additionally, ox-LDL can induce arterial wall inflammation by binding to Toll-like receptors (TLRs), a class of pattern recognition receptors (PRRs) that are widely expressed and elicit pro-inflammatory signals [5]. Finally, under the sustained effect of inflammation, a large number of macrophages accumulate and phagocytose the oxidized lipids, becoming foam cells (Figure 1). This process contributes to the formation of plaques, which can obstruct the blood vessels [6,7]. In addition, the collagen content within plaques decreases throughout the atherogenic process, increasing the risk of plaque instability, resulting in plaque rupture and subsequent thrombosis.

Currently, the surgical treatment of atherosclerosis mainly includes percutaneous coronary intervention [8] and coronary artery bypass grafting [9]. However, these procedures carry the risk of postoperative complications, highlighting the importance of drug therapy as the predominant treatment. Statins such as simvastatin and atorvastatin are the main drugs in the treatment of AS, which function by inhibiting HMG-CoA reductase, a key enzyme involved in cholesterol synthesis, thereby reducing the accumulation of lipids within plaques. Nevertheless, these medications can cause serious side effects in humans, including myalgia, myositis, and gastrointestinal adverse effects [10]. Gene therapy represents an emerging therapeutic approach by delivering nucleic acids into cells to silence target genes [11]. While gene therapy has garnered attention from scientific researchers, challenges remain, such as the poor stability of genes in vivo is and the ineffective uptake by cells [12,13,14,15]. Notably, the significance of early prevention and real-time detection of atherosclerosis is growing due to the inconspicuous nature of early atherosclerosis formation as well as the abruptness of late plaque rupture. However, existing technologies for atherosclerosis prevention and diagnosis are inadequate, with diagnostic techniques often failing to detect the early stages of the disease. Consequently, there is an urgent need to investigate new methods for early detection and prevention strategies of AS.

Nanotechnology, as a potent tool in cargo delivery for disease treatment, diagnosis, and prevention, offers several advantages over conventional therapeutic methods [7]. (i) Targeted delivery: Due to the endothelial dysfunction and increased vascular leakage at the disease site, a similar “EPR effect” occurs at the plaque. Nanocarriers designed with appropriate sizes or engineered by modifying targeting peptides and utilizing biomimetic membranes can passively or actively target the plaque for responsive drug release, offering extended cycle times, enhanced biosafety, and improved therapeutic efficacy [16,17,18,19]. (ii) Improved solubility, stability, and bioavailability. For example, Inclisiran, an siRNA-based drug that has been marketed in Europe, is employed to treat hypercholesterolaemia via semi-annual injections. The nano-delivery system significantly reduces the frequency of dosing compared to other available drugs [20]. (iii) Combination therapies: Combining different drugs delivered into the plaque via carriers can be more effective than monotherapy because different drugs can work synergistically with one another, offsetting each other’s limitations, resulting in advantages such as enhanced efficacy, reduced frequency of dosing, lower overall administered doses, and improved safety. (iv) Multifunctionality: Nanoparticles can be designed to carry multiple functionalities, such as drug delivery, imaging, and targeting, all within a single system. Researchers have developed numerous highly sensitive diagnostic nanomedicines, alongside combined diagnostic and therapeutic nanomedicines, which have yielded promising results.

**Figure 2:**
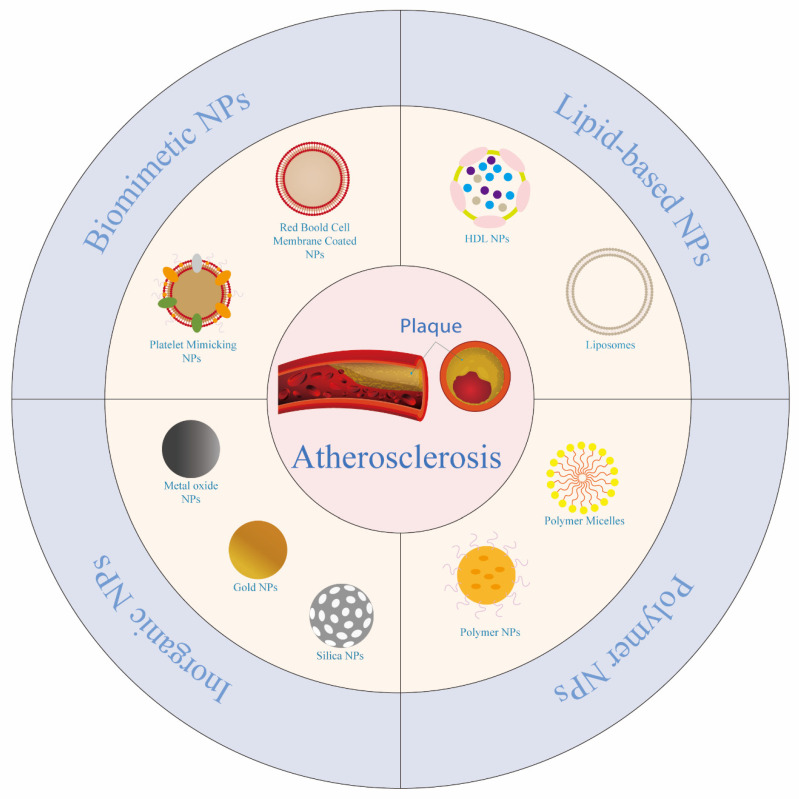
Therapeutic and diagnostic applications of nanoparticles in the management of atherosclerosis.

Currently, numerous reviews have summarized relevant nanoparticles for the treatment of AS, yet there is a paucity of systematic discussions regarding nanocarrier drug delivery for AS. Consequently, this review aims to summarize and categorize the diverse nanoplatform into four main groups based on their unique properties: lipid nanoparticles, polymer nanoparticles, inorganic nanoparticles, and biomimetic nanoparticles. The focus will be on exploring how these nanoparticles enhance the safety and efficacy of drugs, as well as the continuous development and optimization of nanoparticles (Figure 2) made from the same materials.

## 2. Lipid-Based NPs

Lipid nanoparticles, predominantly composed of lipids such as cholesterol and phospholipids, stand as the most extensively researched delivery system for AS. This category encompasses two primary types: liposomes and high-density lipoprotein nanoparticles [7,21,22,23]. Owing to the “like dissolves like” principle, lipid nanoparticles can easily cross the cell membrane, thereby significantly enhancing their cellular uptake. This inherent advantage renders lipid nanoparticles a favored option within the realm of nano-delivery systems.

### 2.1. Liposomes

Liposomes, as lipid-based spherical vesicular systems, are characterized by a lipophilic bilayer situated between two hydrophilic layers [24]. This unique structure enables them to encapsulate hydrophobic agents within the lipid bilayers and enclose hydrophilic agents within the central aqueous compartment, thereby protecting the agents from degradation [25]. Liposomes are notably taken up by cells, which helps to prevent the engulfment of the encapsulated drugs by the mononuclear phagocytic cell system. This process extends the circulation time of drugs in the human body, significantly improves the distribution of small molecule drugs in vivo, and promotes the accumulation of drugs in plaques through the “EPR” effect [7,26]. For instance, Venkatraman et al. developed PEGylated liposomes to deliver the anti-inflammatory drug fluocinolone acetonide (FA). In vivo pharmacokinetics studies demonstrated that the half-life of these liposomes was approximately three times longer than that of free FA, thereby significantly improving the pharmacokinetics of FA. Therapeutically, PEGylated liposomes effectively reduced the number of inflammatory macrophages compared to free FA [27] and they aggregated notably towards the plaque, which could be a beneficial aspect for targeted therapy.

In addition to small molecule drugs, liposomes are also widely used in gene delivery therapy for AS. Ho et al. developed PEGylated cationic liposomes for the delivery of miR-146a, a microRNA known to inhibit the inflammatory response. The results indicated that the miR-146a-lipo group significantly improved the stability and transfection efficiency of miR-146a, therapeutically reducing endothelial and smooth muscle cell activation, pro-inflammatory cytokine release, and foam cell formation compared to the free miR-146a group [28]. This suggests that the PEGylated cationic liposomes are a promising vehicle for enhancing the therapeutic effects of miR-146a in the context of inflammatory diseases.

**Figure 3:**
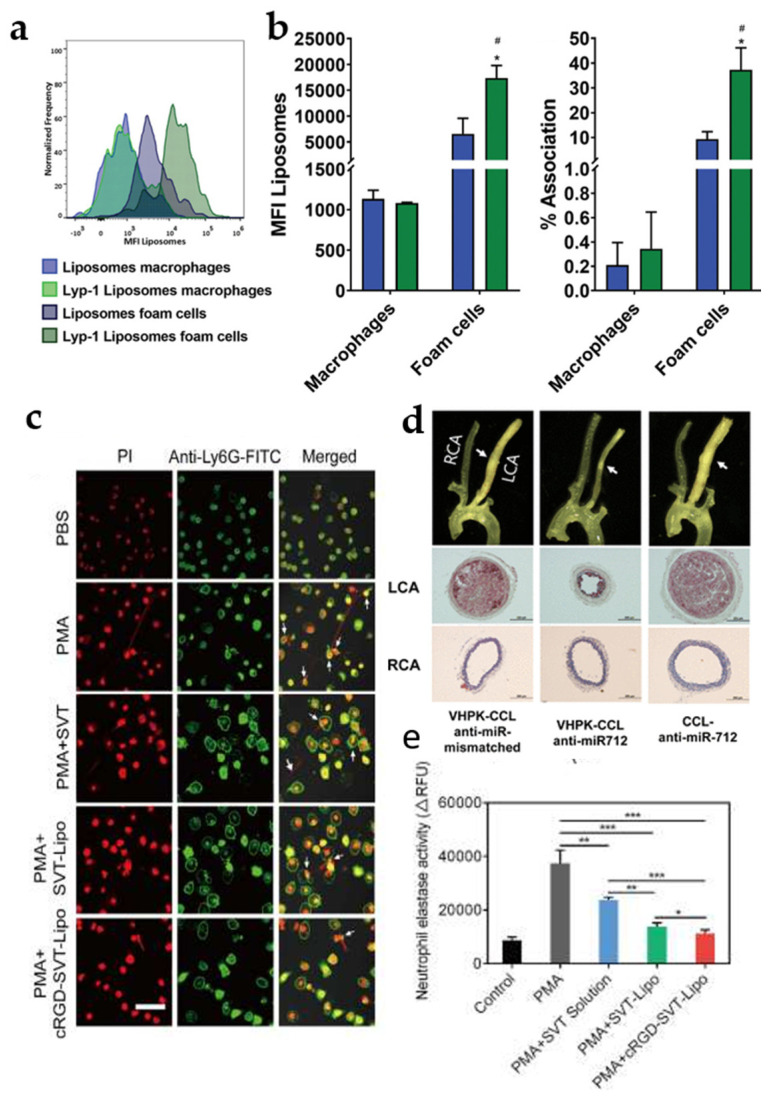
(**a**) Representative MFI plots of targeted versus untargeted liposomes in different cells (macrophages, foam cells). Reprinted from Ref. [29]. (**b**) Mean fluorescence intensity of targeted and nontargeted liposomes in macrophages and foam cells and percentage of positive cells detected by flow cytometry after 2 h of incubation. Graphs show means + SD of three independent experiments; * *p* < 0.05 comparing foam cells to macrophages; ^#^ *p* < 0.05. Reprinted from Ref. [29]. (**c**) CLSM images of neutrophils releasing NET after 4 h incubation with different SVT preparations on the basis of PMA, which was used as a positive and negative control, and PBS. Nuclei are shown in red and NETs in green. Scale bar = 20 μm. Reprinted with permission from Ref. [35]. Copyright 2023 Elsevier. (**d**) Atherosclerosis models were constructed in ApoE^−/−^ mice by ligating the left carotid artery (LCA) and feeding them with a high-fat diet for 2 weeks. The mouse was sacrificed after the treatment and the arterial tree was dissected for bright-field imaging and assessment of histologic findings. Reprinted from Ref. [30]. (**e**) Quantification of neutrophil elastase activity in supernatant after incubation of different SVT preparations with positive neutrophils. Data shown in mean ± SD (n = 3). * *p* < 0.05, ** *p* < 0.01, and *** *p* < 0.001 are considered as significant differences. Reprinted with permission from Ref. [35]. Copyright 2023 Elsevier.

The active targeted modification of liposomes may enhance their accumulation within plaques, whichrepresents a promising approach to improving the therapeutic efficacy of AS. Slütter et al. developed DSPE-PEG2000-Lyp-1 liposomes for the delivery of an LXR agonist (GW3965). These liposomes incorporate LYP-1-targeting peptides, which selectively targets foam cells. Flow cytometry analysis showed a higher uptake of targeted liposomes by foam cells compared to nontargeted liposomes, with little difference between the two uptakes by macrophages. In addition, the proportion of positivity in foam cells was about four times higher for targeted liposomes than for nontargeted liposomes (Figure 3a,b) [29]. This suggests that modifying the targeting peptide indeed improves the targeting of liposomes and enhances their uptake efficiency by foam cells. Jo et al. also developed a similar targeting liposome (VHPK-CCL-anti-miR-712) to deliver anti-miR-712. Results from isolated arterial trees showed that VHPK-CCL-anti-miR-712 significantly inhibited plaque area in mice and that targeted liposomes showed a greater decrease in plaque area compared to untargeted liposomes (Figure 3d) [30]. This is a good indication of the need for targeting.

The excessive production of neutrophil extracellular traps (NETs) by neutrophils has been linked to the progression of atherosclerosis and the rupture of plaques, with neutrophil elastase, a protease released by neutrophils, playing a pivotal role in the formation of NETs [31,32]. The cRGD peptide, which binds to the integrin αvβ3, is selectively expressed on the surfaces of diseased endothelial cells, macrophages, and neutrophils [33,34]. In response to this, Su et al. designed a cRGD peptide-modified neutrophil-hitchhiking liposome (cRGD-SVT-Lipo) to deliver the neutrophil elastase inhibitor sivastigmine sodium (SVT). The potential of cRGD-SVT-Lipo in inhibiting NET release and neutrophil elastase activity was investigated by the authors, who used phosphatidyl myristate (PMA) to stimulate the production of NETs by neutrophils and performed inhibition experiments. The results of the inhibition assay showed that the cRGD-SVT-Lipo group effectively inhibited the formation of NETs to a certain extent and significantly reduced the activity of neutrophil elastase compared with the free SVT group (Figure 3c,e) [35]. Moreover, given that E-selectin is overexpressed on endothelial cells, Li et al. engineered E-selectin-binding peptide-modified liposomes (T-AC-Lipo) to specifically target endothelial cells and deliver atorvastatin calcium (Ato) and curcumin (Cur) to retard atherosclerosis progression [36,37]. Notably, the T-AC-Lipo group minimized plaque area compared to non-targeted liposomes [38]. In summary, with the addition of targeted peptides, liposomes are better able to deliver drugs to macrophages, foam cells, endothelial cells, etc., at the plaque, and the therapeutic effect of the drugs is greatly enhanced.

Aside from targeted peptide technology, biological targeting has seen significant advancement. It is known that macrophages selectively phagocytose apoptotic vesicles from apoptotic cells, mainly due to the increased expression of phosphatidylserine (PtdSer) on the surface of these vesicles [39]. Based on this phenomenon, Ogawa et al. developed PtdSer-modified liposomes encapsulating a peptide-ICG2 (P-ICG2-PS-Lipo). The authors found that mouse macrophages showed higher uptake in PS-targeted liposomes compared to non-targeted liposomes [40]. Building on these studies, Zhang et al. introduced DSPE-PEG2000-cRGDfK based on phosphatidylserine (PtdSer) modification to form a novel liposome (AP-Lipo) for the delivery of pioglitazone (PIO). The authors examined the levels of inflammatory factors in the AP-Lipo group and observed a significant decrease in the levels of IL-1β, IL-6 and TNF-α, which are characteristic M1-type cytokines. Therapeutically, compared to the PIO group, the AP-Lipo group showed several beneficial effects: it significantly increased the number of M2 macrophages, decreased the number of M1 macrophages, lowered the levels of inflammatory factors, and increased the amount of collagen [41]. These findings suggest that the PtdSer-modified liposome not only targets specific cell types but also modulates the immune response and promotes tissue repair, potentially offering a more effective strategy for treating atherosclerosis and related inflammatory diseases.

Interventional therapy is one of the main treatments for cardiovascular disease, with in-stent restenosis (ISR) following bare-metal stent implantation posing a major clinical challenge. To address this issue, Joner et al. employed a liposomal delivery system for prednisolone phosphate, a steroid drug that can inhibit the proliferation of smooth muscle cells in atherosclerosis, to inhibit in-stent restenosis. Notably, the liposomal group demonstrated a significantly reduced percentage of stenosis (22.5 ± 4.4%) compared with the control group (31.0 ± 8.4%) [42].

In the context of atherosclerosis formation, the application of vaccines for prevention holds promise for better outcomes. Negatively charged liposomes, known for their ability to induce humoral immune responses, have emerged as an effective adjuvant delivery system in vaccine formulations, thereby enhancing vaccine efficacy [43]. Sahebkar et al. developed negatively charged liposomes to deliver the immunogenic fusion protein PCSK9-tetanus (IFPT), which targets the low-density lipoprotein receptor (LDLR) that regulates low-density lipoprotein plasma levels [44]. The researchers vaccinated hypercholesterolaemic mice and studied them over time. Analysis of antibody titres showed that the L-IFPTA+ vaccine stimulated a sustained humoral immune response against the PCSK9 peptide and that anti-PCSK9 antibody titres were maximal at 8 weeks. This was accompanied by a significant 42% reduction in LDL-C levels. This showed the potential of negatively charged liposomes to deliver the vaccine. The consensus in immunology is that helper T1 (Th1) CD4^+^ T cells are proatherosclerotic, whereas regulatory T cells and B cells (Tregs and Bregs) are protective against atherosclerosis [45,46,47,48,49]. Evidence from animal and human studies confirms that on a high-fat diet, Treg numbers are reduced or immunosuppressive Treg function is impaired and transformed into pro-atherosclerotic immune cells [50]. Slütter et al. employed negatively charged liposomes (DSPG-lipo) loaded with low-density lipoprotein-derived peptide antigen peptide ApoB1003500-3514 (p3500) to induce antigen-specific regulatory T cells (Tregs). The in vivo pharmacodynamic results showed that the DSPG-p3500 group produced many positive effects compared with the control group: (I) a significant increase in the number of Treg with minimal production of pro-inflammatory factors; (II) a significant reduction in plaque area by about 50% and a significant increase in collagen content; and (III) no significant change in body weight of the mice in the different groups [41]. This suggests that DSPG-p3500 has a good safety profile and that the significant increase in collagen content serves to stabilize the plaques and greatly reduce the risk of plaque rupture. These findings suggest that negatively charged liposomes hold great promise for future research and development in the prevention and treatment of atherosclerosis.

### 2.2. High-Density Lipoprotein (HDL) NPs

High-Density Lipoprotein (HDL), which predominantly consists of phospholipids and the protein apolipoprotein A1 (apoA-1), plays a crucial role in atherogenesis by facilitating reverse cholesterol transport, thereby preventing excessive cholesterol accumulation in macrophages. Therefore, HDL nanoparticles have a wide range of applications in atherosclerosis (Table 1). HDL exists within the body mainly in two structural forms: discoidal and globular. Discoidal HDL (d-HDL) features a phospholipid bilayer, while spherical HDL (s-HDL) consists of a monophospholipid layer and a hydrophobic core [51]. D-HDL is primarily secreted by the liver with subsequent cholesterol uptake and then interacts with plasma lecithin cholesterol acyltransferase (LCAT), which converts d-HDL to s-HDL, which is subsequently translocated to the liver for excretion [52].

HDL possesses a natural targeting advantage because of the mediation of a specific high-affinity clearance receptor BI class (SR-BI) [52,53]. Being an endogenous molecule, HDL can evade phagocytosis by the reticuloendothelial system (RES) [54,55], which plays a key role in the immune response and removal of foreign object. Wu et al. developed two types of HDL particles (TA-d-rHDLs and TA-s-rHDLs). The results of in vitro release experiments showed an increase in the release rate of TA-d-rHDLs from 29.23 ± 2.16% to 43.32 ± 1.67% after the addition of LCAT, which would lead to drug leakage in vivo. Moreover, TA-s-rHDLs were more targeted. This suggests that s-rHDLs are more suitable for drug delivery [52]. Based on the advantages of s-rHDL, Chen et al. developed globular recombinant high-density lipoproteins (s-rHDL) to deliver exogenous GM3. Notably, the therapeutic effect of the low-dose GM3-s-rHDL group was on par with that of the high-dose free GM3 group [22], suggesting s-HDL improves the therapeutic efficacy of GM3 and enhances its biosafety profile.

Compared to spherical recombinant HDL (d-s-HDL), discoidal recombinant HDL (d-r-HDL) is easier to bind to other materials due to its structure. Liu et al. introduced hyaluronic acid-ferrocene (HA-FC), where HA is used to improve specificity and responsive release can be achieved by FC due to the presence of large amounts of ROS in the plaques [56,57,58]. d-r-HDL interacts with the HA-FC-formed host–guest interaction, thus creating a ROS-responsive nano-assembly (HA-Fc/NP3 ST) nanoplatform for delivery of simvastatin (ST). In vivo pharmacodynamics demonstrated a 53% reduction in plaque size, a 63% reduction in plaque lipid deposition, a 52% reduction in serum levels of the inflammatory factor IL-6, a 59% reduction in TNF-α, and a 48% reduction in CCL2 compared to controls [59]. This demonstrated the great potential of d-r-HDL in combination with other materials.

## 3. Polymer NPs

Polymer nanoparticles have emerged as traditional nanocarriers due to their high modularity and amphiphilic character, which enables them to encapsulate hydrophobic drugs effectively. Their versatility has led to their widespread application across various fields. In this section, we will focus on how polymeric nanoparticles can improve drug delivery efficiency as well as safety.

### 3.1. Polymer Micelles

Polymeric micelles are unique structures characterized by a hydrophobic core and a hydrophilic crown, which allows them to self-assemble into nanoparticles when exposed to certain conditions [7]. Their ability makes them particularly useful in the study and treatment of atherosclerosis, where they have been widely investigated for their potential to improve drug delivery and target specific areas of the disease. The excessive uptake of ox-LDL by macrophages through scavenger receptors (SRs) is one of the causes of atherogenesis. To address this problem, Moghe et al. developed glycoskeletal-polyethylene glycol micelles (AMPs), which mimic the hydrophobic properties of ox-LDL and competitively bind to SRs to impede ox-LDL uptake. Notably, the inhibition of ox-LDL was shown to be 85% in the AMPs group compared to the control group, indicating the significant efficacy of glycoskeletal-PEG micelles in inhibiting ox-LDL absorption [60]. Additionally, Moghe et al. also employed AMP-encapsulated ferulic acid derivatives to form micelles (1cM-PFAG), which significantly inhibited ox-LDL uptake and reduced ROS levels compared to the free ferulic acid group [61]. This competitive micelle highlights the importance of micelle structure and provides new directions for researchers.

Gene therapy continues to receive increasing attention from researchers. Research has indicated that nucleotides in a polymeric state can bind to SA receptors on macrophages [62]. Choi et al. demonstrated that nanoparticles coated with DNA could be selectively targeted to macrophages present in plaques [63]. To improve the delivery efficiency of LOX-1 siRNA, Li et al. ingeniously combined and assembled DNA oligonucleotides with ROS-responsive rapamycin prodrugs into micellar PAP-SNAs to deliver LOX-1 siRNA. Notably, in vivo pharmacodynamics showed a 27.5% and 23.3% reduction in plaque area for free Rap and free LOX-1 siRNA, respectively, and a 15.7% and 14.3% reduction for RAP-SNA and LOX-1/RAP-SNA, respectively [64]. This indicated that PAP-SNAs enhance the therapeutic effect of nucleic acids. Due to the great potential of polyelectrolyte composite micelles in delivering gene drugs, Tirrell et al. developed polyelectrolyte composite micelles specifically designed to target inflammatory endothelial cells, with the aim of delivering miR-92a inhibitors to exert anti-inflammatory and antioxidant effects. Importantly, plaque area was substantially reduced in the polyelectrolyte composite micelle group compared to the free miR-92a inhibitor [65]. Thus, polymeric micelles can effectively deliver nucleic acids and enhance their therapeutic efficacy.

**Figure 4:**
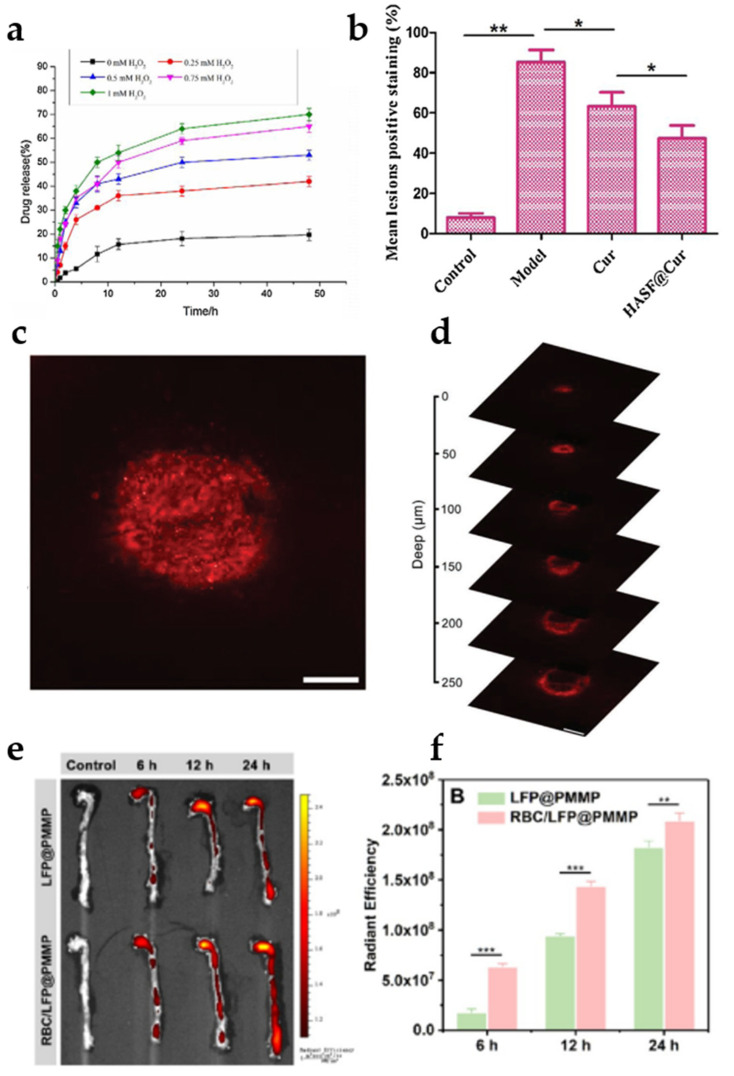
(**a**) ROS-sensitive drug releases in vitro. Data are shown as the mean ± SD (n = 3); * *p* < 0.05. Reprinted with permission from Ref. [68]. Copyright 2019 Elsevier. (**b**) Corresponding percentage of mean lesion positive staining. Data are shown as the mean ± SD (n = 4); * *p* < 0.05, ** *p* < 0.01. Reprinted with permission from Ref. [68]. Copyright 2019 Elsevier. The projection image of atherosclerotic plaque recognized under two-photon CLSM (**c**) and the confocal images of the plaque (**d**) at various imaging depths. The scale bar was 200 µm. Reprinted with permission from Ref. [70]. Copyright 2020 John Wiley and Sons. (**e**) Ex vivo fluorescent images and (**f**) quantitative data of LFP@PMMP and RBC/LFP@PMMP in aortas (n = 6). Reprinted with permission from Ref. [71]. Copyright 2021 American Chemical Society ** *p* < 0.01; *** *p* < 0.001.

During the process of atherogenesis, chronic inflammation triggers the overproduction of reactive oxygen species (ROS), including OH^−^, H_2_O_2_, and O_2^−^_, in the inflamed tissues. Consequently, various polymeric micelles with ROS-sensitive structures, such as thioketal group, ferrocene, and peroxalate ester, have been employed for ROS-responsive drug-targeted delivery [66,67]. For example, Chen et al. developed ROS-responsive polymeric micelles to deliver curcumin. The authors self-assembled polymeric micelles (HASF@Cur micelles) by combining hydrophobic ferrocene, containing ROS-responsive thioketal linkages, with hydrophilic hyaluronic acid. In vitro drug release experiments demonstrated that the cumulative release of the drug increased with rising H_2_O_2_ concentrations (Figure 4a). This showed that HASF@Cur micelles are ROS-responsive and may be released responsively at plaques in vivo. In vivo pharmacodynamics revealed that HASF@Cur micelles produced smaller lesion sizes and exhibited greater efficacy compared to free curcumin (Figure 4b) [68]. This suggests that ROS-responsive polymeric micelles enhance the therapeutic efficacy of the drug. Zhang et al. developed HA-coated ROS-responsive polymeric micelles (SHPEMs), which exhibit ROS-responsive effects through peroxalate bonding. Mice treated with SHPEMs had reduced plaque area, and a more pronounced decrease in ROS levels compared to free drug [69].

The researchers conducted further investigations on polymer micelles with integrated diagnostic and therapeutic properties. For example, Wang et al. utilized the amphiphilic polymer PMPC-PMEMA (PMM) micelles to deliver prednisolone (Pred), an anti-inflammatory drug, conjugated to a two-photon AIE fluorophore (TPP), where the TPP exhibited an ROS-responsive function. The TPP@PMM group effectively inhibited the expression of three inflammatory factors, TNF-α, IL-1β, and MPO, and significantly reduced plaque areas compared with the free Pred group. Notably, after 6 h treatment with TPP@PMM in ApoE^−/−^ mice, fluorescent signals of nanoparticles in the plaques could be observed from different depths (Figure 4c,d) [70]. Wang et al. improved PMPC-PMEMA polymer micelles by introducing erythrocyte membranes, which increased the retention of lipid-specific fluorophores (LFPs) at plaques. Ex vivo aortic fluorescence results showed faster aggregation in atherosclerotic lesions in the RBC/LFP@PMMP group compared to the LFP@PMMP group (Figure 4e,f) [71]. This suggests that RBC membranes can improve the retention time of nanoparticles in plaques.

To quantitatively compare the efficacy of lipid-based nanoparticles and polymeric nanoparticles in atherosclerosis therapy, Alaarg et al. synthesized three types of nanoparticles for the delivery of simvastatin: high-density lipoprotein nanoparticles ( [S]-HDLs), polymeric micelles ( [S]-PMs), and liposomes ( [S]-LIPs) and compared the differences among these formulations. The authors labeled these nanoparticles with ^89^Zr post-injection to assess their in vivo pharmacokinetics and organ accumulation. In vivo pharmacokinetics showed that [S]-PMs and [S]-LIPs exhibited prolonged circulation in the bloodstream for over 24 h compared to [S]-HDLs, with residual ID/g in the blood at approximately 10% and 1%, respectively. All three nanoparticles predominantly accumulated in the spleen and liver, with similar uptake in aortic plaques (~1.5–2% ID/g). Notably, macrophages within the plaques exhibited the highest uptake of [S]-PMs and the lowest uptake of [S]-LIPs, suggesting polymeric micelles may be more readily endocytosed by macrophages. Treatment of ApoE^−/−^ mice with [S]-PMs and [S]-HDLs resulted in a significant reduction in macrophage numbers in aortic plaques compared to the free simvastatin group, whereas the [S]-LIP group showed no significant difference [72]. Consequently, [S]-PMs exhibited the most favorable properties among the tested delivery methods, including the longest circulation time, the greatest ability to target plaque macrophages, and the highest reduction in macrophage numbers. Further research is required to delineate the characteristics of various nanoparticle types, thereby enabling informed decisions regarding the selection of appropriate treatments in AS therapy.

### 3.2. PLGA NPs

Among various nanomaterials, poly (lactic acid-glycolic acid) (PLGA) is an FDA-approved biodegradable and biocompatible copolymer with a long half-life. Thus, PLGA nanoparticles can be used to improve the hydrophobicity, efficacy, and high toxicity of drugs.

The anti-inflammatory and antioxidant properties of curcumin (Cur) give it great potential in the field of atherosclerosis, but it shows poor water solubility and low bioavailability [73,74]. Kumar et al. developed PLGA nanoparticles for the delivery of curcumin (Cur) and a natural bioaugmenter, Bioperine (Bio), which increased the bioavailability of Cur. The nanoparticle group significantly reduced NF-κB, CCL2/MCP-1, CD-36 and STAT-3 activity as well as lowered cholesterol levels compared to the control group [75]. The anti-inflammatory drug colchicine (COL) is not commonly used in clinical treatment due to its severe toxic side effects. Chen et al. developed targeted PLGA nanoparticles (VHPK-PLG A@COL) for the delivery of colchicine (COL) [76]. H&E staining showed no significant histologic changes in the major organs of the mice. Clinical biochemical analyses showed that mice had normal levels of alanine aminotransferase (ALT), alanine aminotransferase (AST), glutamate aminotransferase (CRE), and urea nitrogen (BUN), indicating that the biological functions of the liver and kidney were not affected by systemic administration of the nanoparticles [77].

Imaging agents such as lipid-based staining dyes have safety issues in vivo and are not sensitive enough for more accurate and specific imaging [78]. Zeng et al. employed PLGA nanoparticles (PLGA-DiD-BODIPY) to deliver a lipophilic carbonyl cyanine dye (DiD) and a lipid staining dye (C1-BODIPY 500/510-C12). Researchers fed a high-fat diet to apolipoprotein E knockout (ApoE^−/−^) mice to establish an animal model of obesity. After injection of PLGA NP containing DiD/BODIPY, adipose tissue was harvested and imaged for live cell imaging using confocal microscopy imaging, which demonstrated the ability of DiD/BODIPY to specifically image pro-inflammatory macrophages in vivo with the help of PLGA [79].

PLGA nanoparticles are also effective in improving the safety of drugs, thus enhancing their clinical application. PLGA has bioadhesive properties with the mucosa of the gastrointestinal tract and thus can cross the intestinal epithelium via paracellular and transcellular pathways, thereby enhancing drug absorption in the blood circulation [80,81]. Wang et al. utilized PLGA nanoparticles in order to enhance the oral bioavailability of Lovastatin (LV), and subsequently introduced L-arginine (LA) to further stabilize the PLGA NP formulation. The results showed that the average plasma concentration of LV in C57BL/6 mice was as high as 117.5 ± 56 ng mL^−1^ after 6 h of oral administration, which was higher than that of free LV at 26.16 ± 23 ng mL^−1^ [82]. This suggests that PLGA enhances the oral bioavailability of the drug.

PLGA nanoparticles can serve as drug-eluting scaffolds (DESs) to inhibit in-scaffold restenosis (ISR) due to their high biocompatibility and biosafety. Li et al. developed platelet membrane-encapsulated poly(lactic acid-glycolic acid) (PLGA)/rapamycin (Rapa) composite nanoparticles (PNPs) to PLA (PLA) scaffolds to inhibit in-scaffold restenosis. H&E staining revealed that after one month, the mean neointimal area was significantly reduced in the PNP group (1.19 ± 0.1 mm^2^) compared to the PLA group (2.01 ± 0.1 mm^2^). Similarly, the mean neointimal stenosis ratios were lower (22.89% ± 1.08%) in the PNP group compared to the PLA group (34.44% ± 1.08%), underscoring the substantial inhibitory effect of PNP on stent restenosis [83].

Due to the modifiable nature of PLGA, researchers have introduced targeting agents, enzyme reactants, and biofilm technology to increase the functionality of PLGA nanoparticles. Liu et al. capitalized on the high expression of histatin K (CTSK) in plaques to develop PLGA-PEG nanoparticles (T/R NPs) targeting integrin αvβ3 and CTSK response for the delivery of rapamycin (RAP). The authors found that the T/R NP group demonstrated a prolonged blood retention time compared to the unmodified PLGA nanoparticle (NP) group, significantly enhancing the duration and therapeutic effect of RAP treatment [84]. Karami et al. developed monocyte membrane-encapsulated PLGA nanoparticles for the delivery of the anti-inflammatory drug Gliclazide (GL). Notably, they observed a greater reduction in plaque area and inflammation levels in the NG group, which included monocyte membranes, compared to the NP group without monocyte membranes [85]. Cao et al. introduced macrophage membranes on PLGA to improve specific imaging of IR780. The results of ex vivo aortic vascular imaging showed that IR780 fluoresced more significantly at plaques with the help of PLGA nanoparticles encapsulating macrophage membranes [86]. PLGA can be modified with multiple materials to better enhance drug activity as well as safety. Modified PLGA nanoparticles have great potential in the treatment of AS.

### 3.3. Other Polymer NP

**Figure 5:**
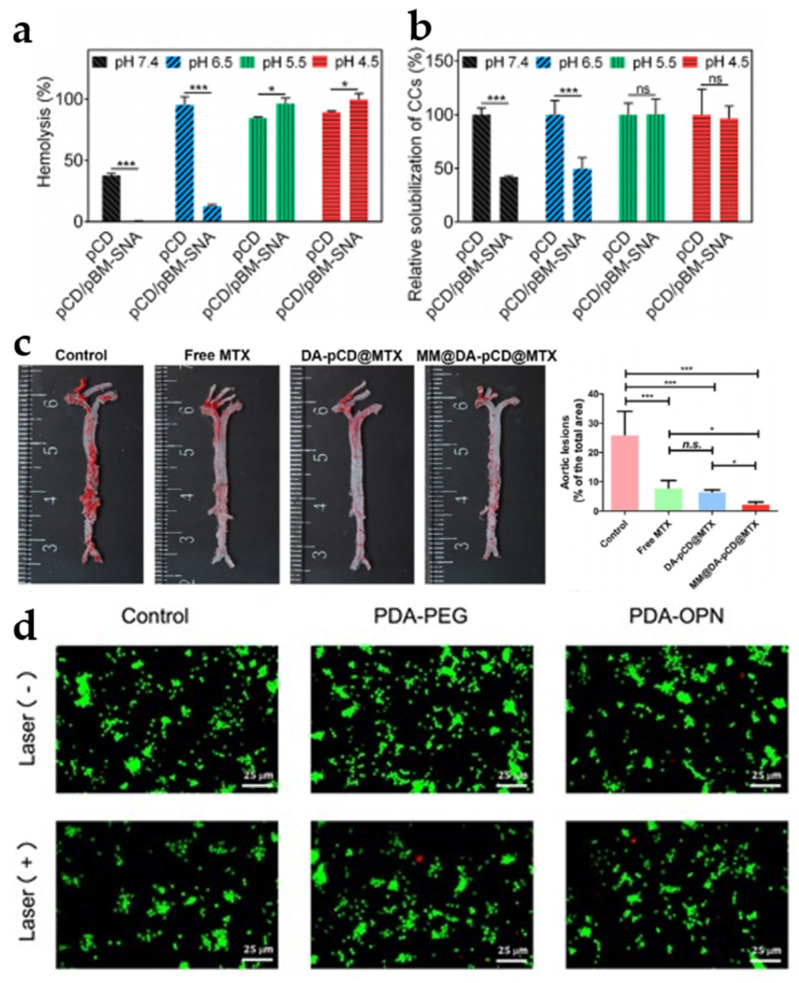
(**a**) Hemolysis of pCD and pCD/pBM-SNA at different pH values (pH 7.4, 6.5, 5.5, and 4.5). Reprinted with permission from Ref. [91]. Copyright 2021 American Chemical Society. (**b**) Relative capacity of CC solubilization of pCD and pCD/pBM-SNA at different pH values (pH 7.4, 6.5, 5.5, and 4.5). Data points represent mean ± SD (n = 3). * *p* < 0.05, *** *p* < 0.001. Reprinted with permission from Ref. [91]. Copyright 2021 American Chemical Society. (**c**) After treatment of ApoE^−/−^ mice with different reagent groups (control, free MTX, DA-pCD@MTX, MM@DA-pCD@MTX), the aorta was isolated and stained with ORO, micrographs of ORO-stained aorta, and their quantitative results (n = 5). * *p* < 0.05, *** *p* < 0.001, and n.s., no significance. Reprinted with permission from Ref. [92]. Copyright 2024 American Chemical Society. (**d**) The different reagents were incubated with foam cells for 4h and then subjected to mild photothermal therapy (irradiation of cells for 10 min (808 nm, 1.0 W/cm^2^)), followed by fluorescent live/dead cell images. Live cells presented green fluorescence, and dead cells showed red staining. Scale bar: 25 μm. Reprinted from Ref. [95].

β-Cyclodextrin (CD), composed of seven (α-1,4-)-linked glucose units with a hydrophobic inner lumen, is ideally suited for encapsulating hydrophobic drugs. Notably, its lumen size is well matched to the size of the cholesterol molecule, enabling it to effectively remove cholesterol from plaques, thus slowing down the progression of atherosclerosis [87,88]. Poly(β-cyclodextrin) (pCD) has been reported to exhibit a superior affinity for hydrophobic molecules [89,90]. Building upon this, Zhang et al. developed poly-β-cyclodextrin (pCD) nanoparticles (pCD/pBM-SNA) to reduce cholesterol levels within plaques. To prevent cyclodextrin uptake from circulating cholesterol, the authors used dextran sulfate (DS) grafted benzimidazole (pBM) as a pH-sensitive switch, in which benzimidazole (pBM) was encapsulated in the cavity and released in the plaque microenvironment to clean the cavity. To test the pH-responsive behavior of the nanoparticles, the authors conducted the hemolysis and the relative solubility of pCD and pCD/pBM-SNA to cholesterol crystals at different pH values (pH 7.4, 6.5, 5.5, and 4.5). The results showed a significant decrease in the solubility capacity and the hemolysis rate of pCD/pBM-SNA at pH 7.4 and 6.5 compared to pCD alone (Figure 5a,b). Furthermore, pCD/pBM-SNA and pCD showed equal solubility and hemolysis rates at pH 5.5 and 4.5. This suggests that pBM acts as a pH regulatory switch that can turn on only in weakly acidic environments. In ApoE^−/−^ mice, pCD/pBM-SNA was effective in reducing plaque area and cholesterol crystal content [91].

To further optimize the polycyclodextrin nanoparticles, Wu et al. introduced macrophage membrane (MM) and dopamine (DA) on the surface of polycyclodextrin (pCD) to form MM@DA-pCD, which was subsequently used to deliver methotrexate (MTX) to induce cholesterol efflux and inhibit foam cell formation. The MM@DA-pCD@MTX possesses multifaceted functions: pCD and MTX work in tandem to address lipid accumulation, with pCD removing the cholesterol crystals and MTX enhancing lipid efflux from foam cells. The results of ORO staining of isolated aorta showed that the plaque area was 24.87% and 7.99% in the control and free MTX groups, and 2.49% in the MM@DA-pCD@MTX group (Figure 5c). In addition, cholesterol image analysis showed that the aortic cholesterol deposition rate was 9.94% in the control group, and the deposited cholesterol was significantly reduced to 7.98% and 6.23% in the MTX and DApCD@MTX NP groups, respectively [92]. This suggests that the therapeutic effect of the free drug was indeed enhanced with the help of polycyclodextrin nanoparticles.

Polydopamine nanoparticles are known for their tunability and biocompatibility, and pH-responsive properties that enable drug release under acidic conditions [93]. Wang et al. developed anti-CD47-modified polyethylene glycol-polydopamine nanoparticles (IBR@PDA-PEG-AntiCD47 (PIP-CD47)) to deliver ibrutinib (IBR) and reduce inflammation at the plaque site. The in vitro drug release assay showed that the cumulative drug release rate of the PIP-CD47 group reached 74.6% within 72 h at pH = 5.4, which was significantly higher than the release rate at pH = 7.4. In response to pH, plaque area was reduced in the polydopamine nanoparticle group compared to the control group, as were the levels of inflammatory factors (IL-1, TNF-*α*, IL-6) [94].

In the context of atherosclerosis, photothermal therapy (PTT) aims to induce foam cell apoptosis to reduce lipid levels in plaque by photothermal heats. Wu et al. developed bone bridging protein-coupled polydopamine (PDA-OPN) nanoparticles to facilitate mild PTT. Fluorescent live/dead cell image results showed that the survival rate of foam cells in the PDA-OPN group remained above 88% after 808 nm laser irradiation (Figure 5d). This indicates that it can indeed function without harming the cells. Oil red O staining experiments showed that the plaque area in the PDA-OPN+ laser group (11.3%) was smaller than that in the control group (24.9%), and the collagen content in the PDA-OPN+ laser group (37.0%) was larger than that in the control group (22.2%) [95]. The rise in collagen content indicates more stable plaques, which laterally suggests that PDA-OPN NPs can treat AS in the context of improving plaque stability.

Poly (beta amino ester) (PBAE),has attracted tremendous attention as a gene delivery vehicle due to its (I) easy surface modification for targeted delivery, (II) ease of synthesis, (III) ability to escape endosomal entrapment, (IV) biodegradability via hydrolytic cleavage of ester groups, (V) low cytotoxicity, and (VI) structural diversity [96,97]. Based on the ease of synthesis and structural diversity of poly (beta amino ester), Jo et al. developed a series of poly (beta amino ester), of which formulations containing 50% C6-K and 50% C6-H (C6-KH polymers) were the most effective in delivering nucleic acids. Subsequently, C6-KH polymers were selected as carriers for the delivery of anti-microRNA-712 (anti-miR-712) to inhibit atherosclerosis. Notably, the levels of miR-712 were decreased with the help of poly (beta amino ester) nanoparticles compared to the control group [98]. Increased levels of inflammation have been reported to stimulate overexpression of VCAM-1, thereby promoting the accumulation of circulating monocytes at the plaque site [99,100]. Therefore, Lehoux et al. developed VHPK-targeting peptide-modified branched poly (beta amino ester) nanoparticles (NP-VHPKs) to deliver a plasmid encoding IL-10, which ultimately reduced VCAM-1 expression and plaque inflammation levels [101].

## 4. Inorganic NPs

Inorganic nanoparticles predominantly include metal oxide nanoparticles and non-metal oxide nanoparticles, such as mesoporous silica nanoparticles, iron oxide nanoparticles, and selenium oxide nanoparticles. This section will provide an in-depth exploration of these distinct inorganic nanoparticles in the treatment of AS.

### 4.1. Metal Oxide NPs

#### 4.1.1. Iron-Based NPs

Plaque instability poses a risk of plaque rupture and thrombosis, which can lead to myocardial infarction (MI) and stroke [102]. The unpredictable nature of plaque rupture underscores the necessity for real-time monitoring of changes at atherosclerotic sites. Therefore, the quest for innovative diagnostic methodologies to detect atherosclerosis has gained urgency.

**Figure 6:**
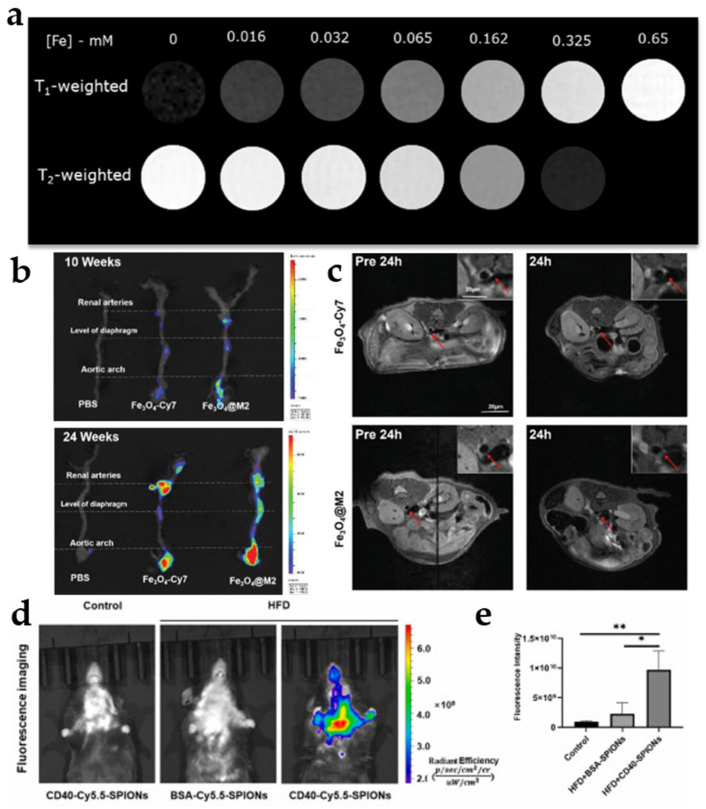
(**a**) In vitro T1- and T2-weighted images. Reprinted with permission from Ref. [107]. Copyright 2017 Elsevier. Ex vivo NIR imaging (**b**) and in vivo T2 MRI (**c**) imaging of the aorta. Reprinted with permission from Ref. [108]. Copyright 2023 John Wiley and Sons. (**d**) Fluorescence imaging of carotid arteries in mice in vivo and quantitative results (**e**). Reprinted with permission from Ref. [109]. Copyright 2023 Elsevier. The analysis among multiple groups was conducted using a one-way analysis of variance test (significant differences: * *p* < 0.05, ** *p* < 0.01).

Iron oxide nanoparticles have emerged as highly promising contrast agents for clinical applications due to their unique magnetic properties, adjustable relaxation rates, high biocompatibility, and facile surface functionalization [103,104]. Iron oxide nanoparticles typically produce negative image contrast due to T2/T2* shortening. Negative image contrast cannot be separated from potential artifactual areas created by signal offset at tissue interfaces, hemorrhages, or water–fat interfaces. T1 shortening avoids this phenomenon by producing positive image contrast [105]. In order to improve iron oxide nanoparticles, Gao et al. developed novel ultra-small iron oxide nanoparticles (Fe_3_O_4_-Cy) as T1 contrast agents. The relaxation rate measurements showed that the longitudinal relaxation rate (r_1_) of Fe_3_O_4_-Cy was 3.52 mM^−1^ s^−1^ and the transverse relaxation rate (r_2_) was 16.84 mM^−1^ s^−1^, and the r_2_/r_1_ ratio was 4.78, which indicated that Fe_3_O_4_-Cy was a T1 contrast agent. In vivo T1 MRI showed a gradual enhancement of the signal emanating from the plaque site within the aortic wall over time, reaching a maximum at 48 h after injection [106]. This suggests that Fe_3_O_4_-Cy is capable of comprehensive plaque imaging and dynamic assessment of its long-term behavior. Whittaker et al. modified magnetic iron oxide nanoparticles (DCION) to develop dual-contrast iron oxide nanoparticles with T1/T2 properties. MRI results show that DCION can shorten T1 relaxation time and shorten T2 relaxation time (Figure 6a). This suggests its potential as a dual positive/negative contrast agent. Reflectance, fluorescence, and superimposed images of damaged carotid arteries showed that DCION2(+) binds strongly and specifically to thrombus but not to normal carotid arteries [107]. This modification of iron oxide nanoparticles solved the limitations of iron oxide nanoparticles to some extent and greatly improved their imaging accuracy.

In addition to changing T2-type iron oxide nanoparticles into T1 contrast agents, the researchers improved the imaging ability of iron oxide nanoparticles by adding targeting agents, combining them with other imaging agents, and other methods. Huang et al. introduced a near-infrared imaging agent (Cy_7_) to develop macrophage membrane-coated Fe_3_O_4_-Cy_7_ nanoparticles (Fe_3_O_4_-Cy_7_@M_2_ NPs). The researchers fed mice with a high-fat diet to form an early lesion model, followed by NIR imaging and MRI experiments. The NIR and MRI results showed that Fe_3_O_4_-Cy_7_@M_2_ NPs were more likely to accumulate at the lesion site compared to Fe_3_O_4_-Cy_7_ NPs (Figure 6b,c) [108]. Side by side, macrophage membranes effectively enhanced the imaging of iron oxide nanoparticles. Zhang et al. also combined MRI with fluorescence to develop CD40-Cy_5.5_ superparamagnetic iron oxide nanoparticles (CD40-Cy_5.5_-SPIONs). Remarkably, the fluorescence signals in the CD40-Cy_5.5_-SPION group were significantly concentrated at the plaque site and the MRI signals in the carotid artery wall were stronger in the mice compared with those in the control group (Figure 6d,e), which may have been due to the high expression of CD40 in the diseased endothelial and macrophages [109]. Therefore, the combination of iron oxide with a variety of materials is also a new direction for the future. Fan et al. utilized the phenomenon that ferricyanide particles with different surface modifiers have different affinities for macrophages to improve the ability to target macrophages. A large number of blue-stained particles were observed in Raw264.7 cells treated with dopamine group (Zn_0.4_Fe_2.6_O_4_-NH_2_) for 2 h, whereas fewer blue-stained particles were observed in the 3,4-dihydroxyhydrocinnamic acid group (Zn_0.4_Fe_2.6_O_4_-COOH) and phosphorylated polyethyleneglycol group (Zn_0.4_Fe_2.6_O_4_-PEG) showed fewer blue-stained particles. This suggests that macrophages prefer to phagocytose the dopamine group, showing side by side that the above theory is correct. In vivo MRI showed an increase in the plaque-to-muscle contrast-to-noise ratio (CNR) over 5–20 min in all three groups, with a much greater increase in the dopamine group than in the other two groups [110]. In addition, the cell survival rate was higher than 80% in all three groups in the concentration range of 0–200 μg/mL, which indicated that the three groups had a good safety profile. In summary, all three groups were able to perform T1-enhanced MRI of atherosclerotic plaques, with the dopamine group having the best results.

#### 4.1.2. Metal Nano-Enzymes

It has been reported that certain metals can be synthesized as nanoenzymes for treating oxidative stress and inflammatory diseases [111,112,113]. Studies indicate that elevated levels of ROS in plaques increase the production of ox-LDL, thereby promoting plaque formation [114]. Therefore, metal nanoenzymes have potential for the treatment of atherosclerosis. Du et al. developed HA-encapsulated cerium dioxide nanoenzymes (HA-CeO_2_ NPs) to scavenge ROS, demonstrating SOD-mimetic and catalase-mimetic activities. HA-CeO_2_ NPs exhibited superior SOD-mimicking activity and significantly reduced ROS levels by WST-8 assay and ROS scavenging assay. Notably, they also possessed self-repair capabilities to sustain ROS depletion. HA-CeO_2_ NPs reduced aortic plaque area by approximately two-thirds and significantly lowered LDL levels in vivo [115].

To further improve the effectiveness of cerium dioxide nanoenzymes in scavenging ROS, Qu et al. introduced gadolinium to prepare a gadolinium-doped CeO_2_ (Gd/CeO_2_) nanoenzymes. Chemical doping such as gadolinium was reported to increase the level of structural defects in cerium dioxide nanoenzymes to enhance their catalytic activity against ROS. The authors added different levels of gadolinium to the cerium dioxide nanoenzymes, and the results showed that the percent Gd content increased from 0% to 7.5%, the CAT-like activity of Gd/CeO_2_ increased from about 50% to about 70%, and the SOD-like activity increased from about 40% to about 75%. It is demonstrated that Gd does enhance the activity of cerium dioxide nanoenzymes. Therapeutically, ex vivo ORO staining of the aorta showed that the Gd/CeO_2_ group significantly reduced the plaque area, decreased the level of inflammatory factors, and increased the collagen content compared to the PBS group [116]. Gadolinium poses a serious risk of toxicity, for which the researchers performed live/dead staining experiments, which showed that the gadolinium/cerium dioxide group had very low toxicity to normal endothelial cells and normal macrophages, with a cell survival rate of more than 80%.

In addition to being able to scavenge ROS, some metal nanoenzymes can be used as imaging agents for magnetic resonance imaging (MRI). Wang et al. chose the T1 contrast agent PCN-222(Mn) (MOF) as a metal nanoenzymes for delivering curcumin (Cur). The MRI in vivo imaging results showed that MOF has good MRI ability. Notably, the interaction of MOF with Cur significantly reduced the plaque area, with the Cur/MOF@DS group showing the greatest reduction in plaque area [117]. In conclusion, metal nanoenzymes with imaging capabilities can synergise with drugs while delivering them to reduce plaque area and are expected to provide early monitoring through MRI applications.

#### 4.1.3. Other Metal Oxide NPs

Abnormal protein phosphorylation and glucose levels contribute to the development of AS and advancements in detection methodologies and tailored therapeutic treatments are important to effectively address these pathogenic elements. Tang et al. developed a Zr-MOF metal framework (PCN-224) for the delivery of iodine (I3-)-Rhodamine B (RhB), which leverages the specific recognition capabilities of Zr (IV) for phosphorylation sites and I3–RhB for glucose. The fluorescence intensity of I3–RhB@PCN224 increased and demonstrated a positive correlation with the concentration range when the nanoparticles were placed in different concentrations of phosphate and glucose. Subsequent detection of fluorescence changes in mouse serum showed that significantly increased levels of glucose and phosphate in the model group compared to normal mice [118]. Similarly, to detect changes in phosphoproteins, Wang et al. exploited the specific interaction between live Zr (IV) and phosphate groups and added porphyrin to reduce background noise and to improve sensitivity [119].

Exogenous gasses, such as hydrogen sulfide (H_2_S) and oxygen (O_2_), have been reported for the treatment of AS. However, their clinical application is hindered by potential side effects, including drug instability and rapid release within the body, which may lead toxicity [120,121,122]. Shen et al. developed DATS-loaded MOC-68-doped polyethylene glycolized MnO_2_ nanoparticles (DATS⊂MOC68@MnO_2_@PEG, DMM), wherein MOC-68 serves as a gas donor carrier for H_2_S-releasing DATS small-molecule drugs, and MnO_2_ participates in a Fenton-like reaction with H_2_O_2_ to release O_2_. In vitro release experiments and cellular experiments showed a significant release of H_2_S and O_2_ in the presence of H_2_O_2_ at pH = 6.5 from DMM. The treatment of DATS and DMM effective inhibited plaque enlargement in ApoE^−/−^ mice and ex vivo ORO staining revealed DMM could significantly reduce the plaque area [123].

### 4.2. Non-Metallic Inorganic NPs

#### 4.2.1. Mesoporous Silica NPs

Mesoporous silica nanoparticles can improve the effectiveness and safety of drugs due to their good biocompatibility, large pore area and small size, and high drug loading capacity [124,125,126]. For instance, Li et al. developed mesoporous silica nanoparticles (MSNs@anti-IL-1β) for the delivery of neutralizing antibodies against interleukin-1β (anti-IL-1β). Compared to the anti-IL-1β group, MSNs@anti-IL-1β group exhibited a more significant reduction in plaque area and better biosafety profile—both of them had similar efficacy in reducing inflammatory factors [127]. In another study, Lu et al. constructed mesoporous silica nanoparticles (IL-1Ra@Cu-MSNs) to deliver copper ions and an IL-1 receptor antagonist (IL-1Ra) to address the problem of hepatic damage of anti-inflammatory agent copper ions (Cu^2+^) and the short half-life of the IL-1Ra. In vitro and in vivo studies showed that IL-1Ra@Cu-MSNs greatly reduced the inflammatory response, plaque area, and macrophage infiltration compared to the Cu-MSNs and IL-1Ra groups, highlighting the enhanced therapeutic effect of mesoporous silica [128]. In conclusion, the exceptional properties of mesoporous silica nanoparticles make them a promising candidate for future applications of AS.

The modification of responsive and targeting agents on the basis of mesoporous silica nanoparticles is of increasing interest. Hyaluronidase and hyaluronic acid (HA), which can target macrophages, have been reported to be overexpressed in plaques. Therefore, Wang et al. developed enzyme-responsive and macrophage-targeting mesoporous silica nanoparticles (SIM@HA-MSN) to address the need for targeted the delivery of simvastatin (SIM). In vitro drug release assays showed a substantial enhancement in the 48-h release rate of SIM upon hyaluronidase exposure. Furthermore, the uptake rate of the HA-MSN was higher than that of the MSN group in LPS-stimulated macrophages while the uptake decreased with the addition of free HA pretreatment, suggesting the targeting efficacy of HA in promoting MSN internalization [129]. On the basis of hyaluronan response, Kim et al. utilized the fact that diseased macrophages overexpress CD9 protein to develop CD9 antibody-modified and hyaluronidase-responsive mesoporous silica nanoparticles (CD9-HMSN@RSV) for the delivery of resuvastatin (RSV), which resulted in significantly reduced cholesterol levels and plaque area compared to free RSA [130].

During the formation of atherosclerotic plaques, the microenvironment becomes mildly acidic due to inflammatory effects, which presents an effective strategy for drug release [131]. Wang et al. exploited the weak acidic response of Schiff base bonds to develop pH-responsive and CD44-targeted dendritic mesoporous silica nanoparticles (H-CuS@DMSN-N=C-HA) for the delivery of anticoagulant drug heparin (Hep) and the photothermal therapeutic agent copper sulfide (CuS). Under 980 nm NIR laser irradiation, the nanoparticles showed a faster and higher increase in temperature with increasing power density and concentration and the photothermal conversion efficiency was 19.57%. A pH-dependent drug release rate of H-CuS@DMSN-N=C-HA was observed for 72 h, with a significant increase in release rate with decreasing pH. Compared to the free Hep group, the H-CuS@DMSN-NQC-HA group exhibited reduced plaque areas, with further reduction observed under NIR irradiation [132]. Wang et al. also developed pH-responsive magnetic mesoporous silica nanoparticles (MMNS@AT-CS-DS) for the delivery of atorvastatin (AT) and photothermite (Fe_3_O_4_). The selective photothermal effect and release of AT at plaques in response to pH resulted in a significant decrease in plaque area under the synergistic effect of both [133].

#### 4.2.2. Quantum Dot NPs

Quantum dot nanoparticles are of interest in atherosclerosis research due to their unique optical properties [134,135,136]. Recently, Smith et al. developed dextran-mimicking quantum dots (Q-Dex) to target macrophages for plaque imaging. Encouragingly, Q-Dex exhibited a higher concentration in plaques and provided clearer imaging at the plaque site compared to dye-labeled Dex probes. However, the insufficient penetration depth of visible light emitted by QD limits its in vivo imaging applications [137]. To address this issue, Yu et al. developed macrophage-derived microvesicle (MMV)-encapsulated dual-emission ZAISe/ZnS quantum dots (ZAISe/ZnS@BSA@MMV), which have visible and infrared light emission sites at 530 nm and 660 nm. Compared to the control, ZAISe/ZnS@BSA@MMV exhibited enhanced accumulation in the plaques in both visible and infrared light [138].

Quantum dots, when combined with other imaging techniques, enhance the accuracy of in vivo imaging. Li et al. introduced MRI by combining carbon quantum dots with superparamagnetic iron oxide. In vivo MRI and fluorescence imaging showed clearer imaging [139]. The excellent gene-loading ability of graphene nanoparticles has attracted the attention of researchers. Zhu et al. combined graphene with quantum dots to form graphene quantum dots (GQDs) to deliver microRNA223. In view of the low toxicity and modifiability of graphene quantum dots, Jiang et al. engineered a multifunctional nanoparticle by initially encapsulating atorvastatin (AT) within graphene oxide quantum dots (GOQDs). This complex was subsequently enveloped in a hybrid membrane derived from macrophage and erythrocyte membranes. Finally, hyaluronic acid (HA) was integrated into this hybrid membrane to yield HA-M@AT@GP. In vitro and ex vivo biological experiments showed a significant decrease in ROS levels and ox-LDL levels, a significant decrease in plaque area, and a significant increase in collagen in diseased macrophages in the HA-M@AT@GP group compared with the free AT group [140].

#### 4.2.3. Other Non-Metallic Inorganic NPs

Selenium nanoparticles with low toxicity and high biocompatibility demonstrate remarkable versatility in modification [141,142]. Furthermore, selenium element is involved in the body’s antioxidant and anti-inflammatory processes. Chen et al. developed mannose-modified selenium nanoparticles (MSeNP@CIP) for the delivery of calpain inhibitory peptide (CIP). The calpain-inhibiting peptide inhibits calpain, thereby increasing ABCA1 activity and enhancing cholesterol efflux. MSeNP@CIP&DiR exhibited stronger fluorescence intensity at the plaque compared to SeNP@CIP&DiRa and the greatest reduction in plaque areas were observed in the MSeNP@CIP group compared to the other groups [143].

Diselenides show powerful functions such as catalyzing the production of NO gas and producing anti-inflammatory and antioxidant capabilities [144]. Selenium and polyphenols have been reported to have coordinated antioxidant efficacy; therefore, Wang et al. developed polyphenol diselenide nanoparticles (CASes). The CASe group showed a stronger H_2_O_2_ scavenging capacity than the other single-drug groups, and that H_2_O_2_ was almost completely scavenged at a concentration of 50 μg/mL in the CASe group. In vivo pharmacodynamics showed a greater decrease in plaque area and ROS levels in the CASe group compared to the single-drug group [145].

Carbon nanocages have received much attention from researchers due to their high drug loading capacity and low toxicity [146]. Cui et al. developed chitosan carbon nanocages (CS-CNCs@Ce6/DS) modified with dextran sulfate (DS) to deliver Chlorin e6 (Ce6). The authors first performed loading rate (LR) and encapsulation rate (EE) measurements, and ultraviolet-visible (UV-vis) spectroscopy showed that LR and EE were 26.1 ± 2.7 and 71.9 ± 3.4 wt%, respectively, which were much higher than those of conventional nanoparticles (liposomes, etc.). This indicates that carbon nanocages have high drug-carrying capacity. Dissection of ApoE^−/−^ mice under PTT and PDT therapies revealed that the CS-CNCs@Ce6/DS group showed the largest decrease in plaque area and H&E staining showed no significant abnormalities in major organs (heart, liver, etc.) [147].

## 5. Biomimetic NPs

This technology uses cells such as leukocytes, erythrocytes, macrophages, and platelets to participate in the preparation of nanoparticles and assist in drug delivery. This technology improves the safety and efficacy of the drug by increasing the targeting of the nanoparticles and prolonging the circulation time of the nanoparticles. (Table 2). Therefore, we will mainly discuss this technology in the following sections.

### 5.1. Erythrocyte Membrane NPs

**Figure 7:**
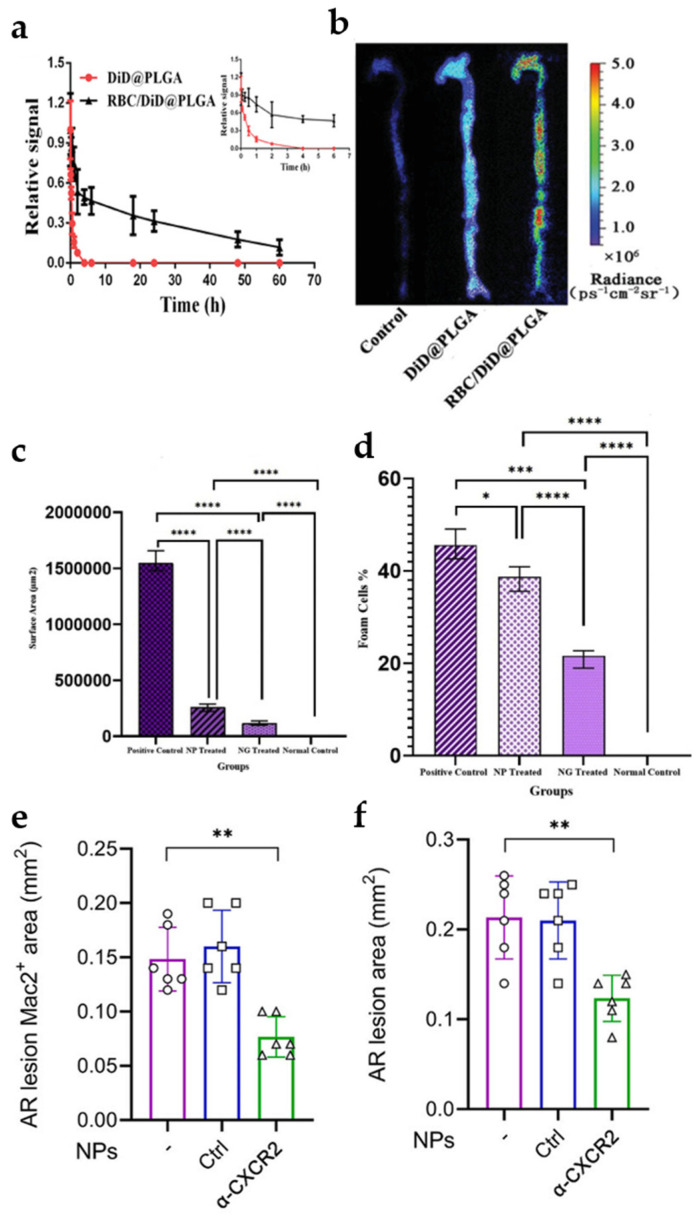
(**a**) Pharmacokinetic studies of RBC/DiD@PLGA and DiD@PLGA in C57BL/6 mice, (n = 5). Reprinted from Ref. [150]. (**b**) Ex vivo fluorescence images of the aorta. Reprinted from Ref. [150]. Results of estimated values of surface area of plaque endothelial area (**c**) and percentage (**d**) of total foam cells (n = 3). * *p* < 0.05, *** *p* < 0.001 and **** *p* < 0.0001. Reprinted with permission from Ref. [151]. Copyright 2023 Elsevier. Aortic root (AR) lesion size (**e**) and measurements (**f**) of Mac^2+^ areas in AR lesions, (n = 6). ** *p* < 0.01. Reprinted with permission from Ref. [161]. Copyright 2023 Elsevier.

Red blood cells are the cells that grant nanoparticles immune evasion and longevity in circulation [148,149]. In a specific study, Wang et al. engineered erythrocyte membrane-coated poly(lactic-co-glycolic acid) (PLGA) nanoparticles (RBC/RAP@PLGA) for the delivery of rapamycin (RAP). In vivo pharmacokinetics showed that the total retention of RBC@PLGA and DiD@PLGA nanoparticles in the blood was approximately 31% and 17% at 24 and 48 h after injection, respectively (Figure 7a). Fluorescence imaging of isolated aorta showed significant aggregation of RBC/DiD@PLGA in atherosclerotic plaque areas (Figure 7b) [150]. Erythrocyte membrane technology does improve the long circulation time of nanoparticles and their retention in plaques. Akrami et al. also delivered anti-inflammatory microsome glibenclamide using a similar approach. H&D staining showed that the erythrocyte membrane-encapsulated PLGA nanoparticles group showed an approximate 13.24-fold reduction in plaque area (Figure 7c) and a 2.1-fold reduction in the total number of foam cells (Figure 7d) as compared to the positive control group [151]. Probucol (PU) is a promising anti-atherosclerotic drug, but its difficult absorption in the gastrointestinal tract and insufficient bioavailability have led to its poor therapeutic efficacy [152,153]. Zeng et al. also utilized a similar method to deliver PU. In terms of pharmacokinetics, the terminal elimination half-life (t1/2z) of RP-PU was significantly better than that of PU. This suggests that the vector significantly increases the bioavailability of PU. In terms of treatment, RP-PU is more effective than PU in reducing plaque area and ROS level [154]. Therefore, erythrocyte membranes seem to enhance the effectiveness of drugs in the treatment of atherosclerosis.

The combination of erythrocyte membranes with other materials is increasingly showing great potential [155,156,157,158,159]. For example, Sun et al. combined erythrocyte membranes with platelet membranes to form erythrocyte–platelet membrane-encapsulated nanoparticles ([RBC-P]NPs) for the delivery of anti-CXCR2 antibodies (CXCR2 is a receptor for activated platelets to attract circulating monocytes). The hybrid membrane has both inflammatory tropism and long-circulating capacity [149,158,160]. Confocal fluorescence imaging showed optimal targeting of [RBC-P]NPs to activated monocytes compared to RBCNPs. Targeting was indeed improved after the fusion of platelet membranes. Therapeutically, anti-CXCR2 [RBC-P]NP-treated mice had a 41% reduction in plaque area (Figure 7e) and a 51% reduction in Mac^2+^ macrophage content (Figure 7f) compared with controls [161]. This suggests that hybrid membranes have great potential.

The change from low to high shear stress in the body plays an important role in the formation of atherosclerosis, and it is often at sites of high shear stress that atherosclerosis tends to occur [162,163]. So, Li et al. introduced dual-responsive dendrimer molecules (SA PAM) to form du-al-responsive dendrimer nanoparticles (SA PAM@RBCs) on the basis of erythrocyte membranes. To investigate whether there is a shear stress response in SA PAM@RBC, the authors developed an in vitro shear microfluidic circulatory system for in vitro shear stress response experiments. The experimental results showed that at 100 dynes/cm^2^, the green fluorescence signal of SA PAM was significantly attenuated and the green/red fluorescence ratio was significantly decreased. This demonstrated that SA PAM could be shed from erythrocytes in large quantities. This proved its massive shedding from RBCs in large quantities. Meanwhile, at 20 dynes/cm^2^, its green fluorescence and red fluorescence co-localized and the green/red fluorescence ratio was consistent with that of the control [156]. This suggests that in healthy blood vessels, it does not release. In conclusion, the combination of erythrocyte membranes and other nanomaterials to achieve passive and active targeted delivery is one future trend.

### 5.2. Platelet Membrane NPs

Damaged vessels during atherogenesis stimulate platelet adhesion and enrichment, suggesting that platelet membranes have natural targeting advantages in addition to the functions of erythrocyte membranes [164,165]. So, platelet-related technologies have great potential. For example, Liu et al. performed cellular uptake experiments in order to assess the targeting properties of platelet membranes. The results showed that in foam cells, the nanoparticle group with encapsulated platelet membrane showed a strong red fluorescent signal whereas the group without platelet membranes showed almost no red fluorescent signal in foam cells [166]. Platelet membrane-encapsulated nanoparticles with specific inflammatory targeting have great potential in the treatment of atherosclerosis.

Commonly used therapeutic and monitoring agents have problems in vivo such as insufficient bioavailability, hydrophobicity, severe adverse effects, and poor targeting, while biofilm technologies (platelet membranes, etc.) can improve their bioavailability, biosafety, targeting, etc. [7,167] Wang et al. also took advantage of the natural targeting properties of platelet membranes by encapsulating mesoporous silica nanoparticles within them to deliver anti-CD47 antibodies. The results of oil red O staining showed that com-pared with the control group (46.7 2%), the plaque area of aCD47, aCD47@MSN, and aCD47@PMSN decreased to 43.5 ± 2.1%, 41.6 ± 1.6%, and 16.6 ± 1.7%, respectively [168]. Fontana et al. utilized the platelet membrane wrapping technique to deliver the hydrophobic drug curcumin (Curc) (Curc@Lignin@TA@PL). The results of cytotoxicity experiments showed that NPs remained safe for normal macrophages at concentrations up to 50 μg mL^−1^ [169]. Liu et al. also delivered photoresponsive NO prodrugs (RBT-NO) using the platelet membrane wrapping technique, which releases NO for endothelial repair at the plaque site. The H&E slice results showed that the control group had signs of thrombosis, such as thickening of the vessel wall, smaller lumen, and thickening of the intima, whereas the treatment group had a regular arrangement of the vessel wall tissue and a significant improvement in thrombus accumulation [170]. Fu et al. designed ROS-responsive nanoparticles (PGA@PMP) coated with platelet membranes to enhance the tar-geting efficiency of photoacoustic π probes and the immunomodulatory complex GWAS. Notably, PGA@PMP can induce an increase in the number of M2 macrophages as well as regulatory T (Treg) cells to rebuild the immune microenvironment [171].

Gene therapy has attracted more and more attention from scientific researchers, but there are problems such as low delivery efficiency and the poor stability of genes [172]. Liu et al. utilized platelet membranes wrapped around nanobubbles and assisted ultrasound-targeted microbubble disruption (UTMD) to deliver Nox2 siRNA (PNBs-siNox2). Notably, in vivo pharmacodynamics demonstrated a significant reduction in plaque area to 31.80 ± 2.32% (*p* < 0.05) in the PNBs-siNox2 group compared to the PBS group, and a significant reduction in plaque area to 19.78 ± 1.45% (*p* < 0.05) in the PNBs-siNox2 + US group [173]. Platelet membrane technology protects the gene drug well so that it can function.

### 5.3. Macrophage Membrane NPs

**Figure 8:**
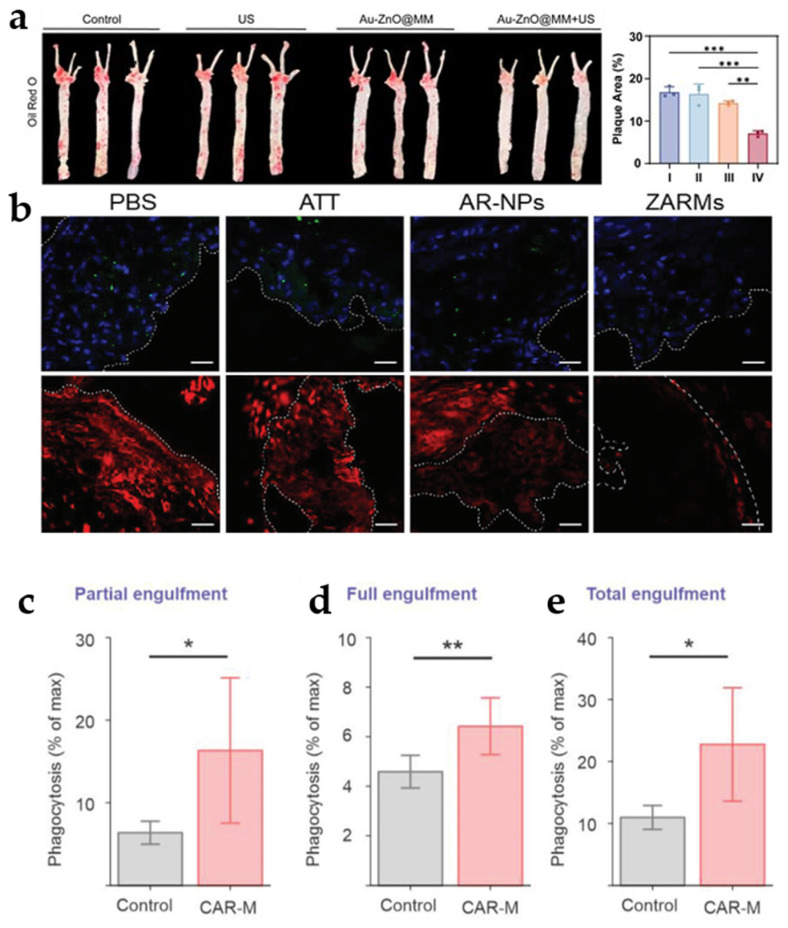
(**a**) Representative photograph of ORO-stained aorta and quantitative statistics. ** *p* < 0.01, *** *p* < 0.001. Reprinted with permission from Ref. [183]. Copyright 2024 John Wiley and Sons. (**b**) CD47 and caspase-3 immunofluorescence images. CD47 in red and caspase-3 in green. Reprinted with permission from Ref. [185]. Copyright 2024 John Wiley and Sons. Quantification of (**c**) partial, (**d**) full, and (**e**) total internalization of standard ACs by either M1 control macrophages or CAR-Ms by CellTagging. Data are mean ± SEM; n = 3; * *p* < 0.05, ** *p* < 0.01 by one-way ANOVA with Tukey post hoc analysis. Reprinted from Ref. [188].

Macrophages play a vital role in the various stages of atherosclerosis (formation–expansion–necrosis) [174,175,176]. Depending on their phenotype and function, macrophages can generally be divided into M1 (pro-inflammatory) and M2 (anti-inflammatory) types [177]. M1 macrophages have been reported to migrate to the inflammation site by targeting VCAM1 and ICAM1, which are overexpressed on inflamed blood vessels and activated smooth muscle cells [177,178,179,180]. Thus, Wu et al. constructed an M1 macrophage membrane-encapsulated nanomedicine (MM@CD-PBA-RVT) to deliver Resveratrol (RVT). The researchers first performed uptake experiments with normal macrophages. Flow cytometry analysis showed that the calculated fluorescence uptake of CD/DiD was approximately 1.2-, 8.3-, 2.6-, and 20-fold higher than that of MM@CD/DiD at 0.5, 1, 2, and 4 h, respectively [181]. This suggests that M1 macrophage membranes have a great advantage in immune escape. Wu et al. used a similar approach to deliver methotrexate (MTX), using macrophage “homing” delivery to significantly promote cholesterol efflux to reduce the cholesterol burden on macrophages [182]. Cheng et al. coated macrophage membrane (MM) onto the surface of rod-shaped Au-ZnO schottky junction to form nano-particles (AuZnO@MM) to effectively alleviate the progress of atherosclerosis. The ORO staining results showed that the Au-ZnO@MM group had the lowest mean percentage of plaque area of 7.07% (Figure 8a), which was much lower than that of the control group (16.83%) [183].

Foam cells accumulate excess lipids and then form apoptotic cells (AC), which ultimately trigger secondary necrosis and chronic inflammation. Studies have shown that upregulation of CD47 receptors on the surface of apoptotic cells inhibits the removal and exocytosis of this cell during apoptosis [184]. Guo et al. used macrophage membranes to form ZARMs to deliver drugs that inhibit CD47. CD47 immunofluorescence staining showed that the ZARMs group had the lowest CD47 red fluorescence signal, and Caspa-se-3 immunofluorescence showed a gradual decrease in the number of apoptotic cells (Figure 8b) [185]. This suggests that apoptotic cells are indeed removed by decreasing CD47 levels. CD47 blockers promote macrophage uptake of apoptotic cells after excessive blockade of CD47, which increases the cholesterol burden on macrophages, leading to the formation of cholesterol crystals (CCs) and CD47 blockers are currently associated with side effects such as anemia [184,186,187]. Lee et al. developed chimeric antigen receptor macrophages (CAR-Ms), which target and phagocytose anti-phagocytic apoptotic cells expressing CD47. Subsequently, the authors attached modified HPβ-CD lipid nanoparticles (β-CD LNP) to the surface of CAR-M to form CAR-M/β-CD LNP. Anti-CD47 CAR-M clears apoptotic cells. Phagocytosis experiments showed 6.42 ± 1.15% and 16.3 ± 8.81% of M1 CAR-Ms partaking in full and partial engulfment, respectively, compared to 4.59 ± 0.66% and 6.37 ± 1.39% of M1 control macrophages engaged in such processes (Figure 8c–e). CAR-T macrophages efficiently phagocytosed apoptotic cells and showed a 2.39-fold and 2.03-fold increase in Abca1 and Abcg1 expression, respectively [188]. CRT-M showed great potential in the treatment of AS.

In addition to its targeting properties, M2-type macrophages have anti-inflammatory effects of their own. Consequently, Zhang et al. fused the M2 macrophage membrane with lipopeptide (DOPE-pp-HBSP) to form mixed nanovesicles (MLPNVs) for the delivery of simvastatin (ST). In terms of treatment, plaque area was significantly reduced and much smaller in the ST@MLP NVs group compared to the free ST group. In addition, the level of pro-inflammatory factors released by macrophages in plaques in the MLPNVs group decreased [175]. This suggests that M2 macrophage membranes reduce the level of inflammatory factors and make drug treatment more effective. Furthermore, macrophage membranes have shown good results in delivering diagnostic imaging nanoparticles [189]. To further improve their targeting, Xiang et al. combined platelet membranes (PMVs) with macrophage-derived extracellular vesicles (M2EVs) to form P-M2EV membranes to deliver miR-99a-5p, an miRNA that inhibits foam cell formation. Inverted fluorescence microscopy showed a significantly higher uptake of P-M2EV by foam cells than M2EV, and flow cytometry demonstrated these results. Reacting to the treatment, the P-M2EV group significantly inhibited foam cell formation in vitro and significantly reduced plaque area in vivo [190].

### 5.4. Leukocyte Membrane NPs

Leukocytes and neutrophils are cells involved in the inflammatory process in vivo, and they target inflammation by binding to vascular cell adhesion molecule 1 (VCAM-1) and intercellular adhesion molecule-1 (ICAM-1) on inflamed endothelial cells [191,192]. Based on this property, leukocytes and neutrophils have potential in the treatment of AS. Chu et al. used neutrophil membranes to deliver the zeolitic imidazolate framework-8 (ZIF-8) “core” (AM@ZIF@NM) encapsulating microRNA-155. Confocal fluorescence images showed strong red fluorescence in the Cy5-AM@ZIF@NM group in inflammatory endothelial cells and weak red fluorescence in the Cy5-AM@ZIF@NM group in normal endothelial cells. Ex vivo imaging showed significantly higher fluorescence in isolated aortic tissue from mice with Cy5.5-AM@ZIF@NM NPs compared to the Cy5.5-AM@ZIF@NM NPs group. This showed that neutrophil membranes are inflammation-targeted [191]. In conclusion, the incorporation of leukocyte membranes enhanced the targeting ability of the nanoparticles, thereby concentrating more drugs onto the plaques and improving the efficacy.

Except for the delivery of therapeutic agents, Xie et al. developed a leukocyte membrane encapsulating simvastatin (ST) and adsorbing targeted apolipoprotein A-I-mimicking 4F peptide (AP) to deliver Fe_3_O_4_ magnetic nanoclusters (MNCs) (MNC@M-ST/AP). The authors established an in vitro Transwell migration assay and both CLSM imaging and time-dependent ICP measurements showed that the MNC@M-AP+LPS group displayed higher foam cell uptake efficiency and migration capacity than the MNC@M+LPS group, suggesting a further increase in leukocyte membrane targeting in the presence of AP. T2-weighted MRI and ex vivo fluorescence imaging validated the targeting performance of the MNC@M-ST/AP group, and mice treated in the MNC@M-ST/AP group showed the most pronounced T2-weighted signals and average fluorescence intensity in the plaque region. In terms of in vivo treatment, compared to the PBS group, Aortic lesion area was reduced by 72.3 per cent in the MNC@M-ST/AP group [193]. Combining other nanomaterials with leukocyte membranes can further enhance the therapeutic effect of nanoparticles, and thus has great potential.

## 6. Summary and Perspective

Atherosclerosis, a chronic disease regulated by immune and lipid metabolic processes, significantly contributes to cardiovascular disease. Currently, many drugs used to treat atherosclerosis exhibit low efficacy and pronounced side effects. With steady advancement in nanocarrier technology, nanoparticles are now leveraged to enhance the delivery and efficiency of drugs and diagnostic agents. Due to endothelial dysfunction and increased vascular leakage, the atherosclerotic microenvironment produces a situation similar to a tumor that exhibits EPR-like profiles. The nano-delivery systems, through specific design and functional modification, can achieve precise positioning and sustained release of drugs at AS lesion sites, thereby improving treatment outcomes and reducing systemic adverse reactions.

Up to now, only a few nanoparticles have been reported in clinical trials for human use. For example, Van der Valk et al. employed long-circulating PEG liposomes to deliver prednisolone to patients with atherosclerosis. The circulating half-life (t^1^/_2_) of these PEG liposomes was 7 to 15 times longer that of free prednisolone. Additionally, 75% of the macrophages isolated from patient plaques contained liposomes. DCE-MRI results showed that LN-PLP does not decrease the kinetic parameter K^trans^, which suggests no significant improvement in arterial wall permeability. Lipid levels and inflammation levels did not exhibit a significant change either before or after treatment [194]. Additionally, Kharlamov et al. employed silica–gold nanoparticles to perform plasma photothermal therapy (PPTT) on patients. Compared to conventional stents interventions, patients treated with PPTT exhibited a reduction in atherosclerotic plaque volume and necrotic cores, enhanced coronary vasodilatation, and decreased cardiovascular mortality [195]. Nanoparticles are exhibiting extensive utility in atherosclerosis management for humans through diverse applications such as targeted drug delivery, advanced medical imaging, anti-inflammatory interventions, biomimetic therapies, and stimuli-responsive treatments. This versatility will introduce novel strategies and methodologies for the treatment of AS in humans, despite the numerous challenges that hinder the clinical translation of nanoparticles.

Additionally, artificial intelligence (AI) is being employed to transform traditional nanoparticle design methods, which typically rely on time-consuming and expensive trial-and-error processes. In the field of cardiovascular research, the application of AI is expected to significantly enhance the design efficiency, therapeutic efficacy, and safety of nanoparticles [196,197,198,199]. Specifically, AI in the design of cardiovascular disease nanoparticles can be summarized from the following aspects: (i) Data-driven material selection: AI can analyze extensive biomedical data to predict the physicochemical properties of different materials, such as the stability, biocompatibility, and ability of nanoparticles to penetrate biological barriers. This will assist researchers in selecting the most suitable materials, thereby improving the performance of nanoparticles. (ii) Optimization of synthesis processes: AI can predict outcomes under different synthesis conditions, thereby identifying the most effective synthesis pathways. This approach can increase the yield and purity of nanoparticles while reducing production costs. (iii) Surface functionalization guidance: AI can predict the binding capabilities and biological activities of different ligands, thereby guiding the surface functionalization of nanoparticles to enhance their targeting capabilities and therapeutic effects. (iv) Toxicity and biodistribution assessment: AI can analyze extensive experimental data and clinical research to predict the behavior of nanoparticles in the body, including their toxicity and biodistribution, ensuring their safety and efficacy.

Hybridized nanoparticles can combine the properties of different materials and utilize the advantages of different materials to improve drug delivery efficiency. Sanchez-Gaytan et al. used apolipoprotein-coated PLGA nanoparticles to form novel HDL-like nanoparticles (PLGA-HDL). Significant cholesterol efflux capacity of PLGA-HDL was observed in all cell lines. NIRF imaging of isolated aortas showed that DiR-labeled PLGA-HDL was predominantly concentrated at plaques, and in addition, at the cellular level, PLGA-HDL co-localized with macrophage (CD68^+^) staining in plaques [200]. This suggests that PLGA-HDL enhances the accumulation of nanoparticles in macrophages in plaques, providing a delivery platform for subsequent encapsulated drugs.

Lu et al. used a polyethyleneimine (PEI) condensed core for electrostatic adsorption of anti-miR155, followed by wrapping an ApoA-1-modified lipid bilayer coating around the outer layer to form polymer–lipid hybrid high-density lipoprotein mimetic nanoparticles (PEI/HNPs). ApoA-1 in the outer coating resulted in approximately 2.1-fold higher macrophage uptake of PEI/HNPs compared to non-targeted PEI/Lipo. qRT-PCR detected significantly lower miR155 expression in vitro in the PEI/HNP group compared to the PEI group. The results of intracellular ROS levels and cholesterol efflux showed that the PEI/HNP group significantly reduced ROS levels and had the strongest cholesterol efflux ability [201]. This suggests that hybridized nanoparticles can take advantage of the strengths between different nanoparticles to improve drug delivery and therapeutic efficiency.

However, there are still challenges to overcome.

Firstly, atherosclerosis usually occurs at sites of high arterial blood flow and in high shear environments, and nanoparticles are easily damaged, leading to uneven or premature drug release, thereby affecting the treatment outcome. Additionally, nanoparticles have a short circulation time in the blood and are easily cleared by the immune system, making it difficult to effectively reach the lesion site. So, to fix this, researchers often encapsulate nanoparticles with biological membranes such as red blood cell membranes and macrophage membranes to maintain their stability in high shear environments. At the same time, by optimizing the chemical structure and surface functionalization of nanoparticles, such as introducing pH-sensitive or oxidative stress-responsive groups, their targeting and therapeutic specificity in specific pathological environments can be further enhanced.

Secondly, there are biocompatibility and safety issues. Although nanodelivery systems have many advantages, their long-term safety and biocompatibility still require further research. Some nanomaterials may cause immune or toxic reactions, affecting the treatment outcome. Liposomes with a positive charge are susceptible to recognition by immune cells (macrophages, etc.), potentially triggering inflammation within the body. Utilizing neutral lipids or ionizable lipids to construct liposomes may serve as an effective solution to address these concerns. In addition, both HDL nanoparticles and liposomes face the challenge of premature leakage in vivo, which underscores the need for heightened attention to the off-target distribution of drugs. This can lead to adverse side effects, necessitating careful consideration of drug delivery strategies to minimize these risks.

The in vivo safety of inorganic nanoparticles is a critical consideration in their application as therapeutic agents. These nanoparticles, such as iron oxide nanoparticles, exhibit limited biodegradability, which raises concerns about their long-term persistence in the body to induce toxic reactions and immune responses. Furthermore, the intricate metabolic pathways of these materials are not well understood, complicating the assessment of their potential toxicity. It is imperative that future research focuses on delineating the in vivo behavior and degradation pathways of these nanoparticles to ensure their safe and effective use in therapeutic applications.

The in vivo toxicity of polymeric nanoparticles is primarily associated with their material composition. Cationic materials, for instance, have the propensity to stimulate the immune system and ultimately activate inflammation. Additionally, materials that are poorly degradable can pose metabolic challenges in vivo and may induce apoptosis, thereby contributing to toxicity. It is also noteworthy that materials may interact with proteins in vivo or be affected by changes in pH or temperature, which may result in structural or material alterations. Furthermore, the in vivo stability of the polymeric micelles is a concern, as they may dissociate prematurely at low concentrations, potentially affecting their therapeutic efficacy.

Bionanoparticles possess the capability to evade the immune system and avoid binding to serum protein, thereby preventing nanoparticle aggregation. However, the use of exogenous cell membranes in vivo can lead to pitfalls, such as immune stimulation. Moreover, there are safety concerns related to the encapsulation of nanoparticles using cell membrane technology, necessitating careful consideration and further research to mitigate potential risks. Recently, carrier-free nanoparticles have garnered significant attention from researchers due to their high drug loading capacity and ease of preparation. These advantages make them particularly promising in the treatment and diagnosis of AS. High-density lipoprotein (HDL) nanoparticles have a natural advantage in the treatment of atherosclerosis, and there is a need to take full advantage of other nanocarriers in the future and combine their structural properties with those of HDL nanoparticles.

Thirdly, there is the question of how to weaken the barriers to clinical applications of nanoparticles in AS. The primary barriers to the clinical translation of most nanoparticles include challenges in large-scale industrialization, in vivo toxicity issues, suboptimal in vivo metabolism, and insufficient in vivo stability [202,203]. For example, liposomes face limitations due to their low encapsulation efficiency and instability, while the preparation process of polymer nanoparticles is often complex, hindering large-scale production. Inorganic nanoparticles exhibit toxicity issues and lack biodegradability, and the intricate preparation of biomimetic nanoparticles similarly prevent their large-scale industrialization.

Regulatory barriers are a major obstacle to the clinical translation of nanotechnology. Nanoparticles, as multi-component structures, necessitate meticulous analysis under Good Manufacturing Practice (GMP) conditions and the establishment of reproducible manufacturing processes to guarantee consistent product quality. This complexity presents a challenge for regulatory approval, as established criteria for evaluating nanoparticle-based drugs are often lacking or insufficient. To navigate this challenge, it is advisable to collaborate closely with regulatory authorities from the early stages of development to meet the necessary requirements and expedite the development process.

Scalability emerges as another pivotal barrier in the translation of nanotechnology from the laboratory to large-scale manufacturing. The process of nanomedicine manufacturing usually involves several complex steps, and subtle changes in the preparation process may significantly affect their physicochemical properties (e.g., size, shape, composition, crystallinity, drug loading, drug release, surface functionality, etc.). The transition must be executed without compromising the quality and uniformity of the nanoparticles, a task that is inherently complex that requires meticulous attention to detail and robust manufacturing techniques.

Clinical translation of nanoparticles is an expensive and time-consuming process. The complexity of the dosage form design during preparation can increase the cost and time of preparation. Over-regulation may be necessary to ensure the safety and quality of nanoparticles, whereas over-regulation can increase the cost of obtaining regulatory approval and/or consume a significant portion of the patent life. From a commercial perspective, most pharmaceutical companies do not currently have a good grasp of the necessary infrastructure required for commercial development of nanoparticles, and these require significant cost and time to build a basic framework.

The carrier-free nanoparticles can reduce reliance on complex equipment and high-cost materials, thereby lowering the overall cost. Moreover, during the development of atherosclerosis, large amounts of ROS and enzymes such as hyaluronidase are produced at plaque sites. Researchers have developed a series of responsive nanosystems based on these characteristics, achieving good results. As plaque sites contain large amounts of lipids, lipid-specific probes have shown great potential in diagnosing atherosclerosis, providing significant imaging effects and characteristic fluorescence in lipid-rich cells. In addition, combining different response modalities is also an effective approach.

Last but not least, nanocarriers face many biological challenges that still need to be worked out. One of the major challenges encountered by nanocarriers is the immune response, which can be significantly influenced by the surface charge of the nanoparticles. Specifically, positively charged nanoparticles may induce phagocytosis by immune cells, potentially leading to inflammation. To mitigate this issue, it is imperative to utilize nanoparticles with a neutrally or negatively charge, employ biomimetic cell membrane technology, or modify the nanoparticles with PEG. These strategies are aimed at reducing the immunogenicity and enhancing the biocompatibility of nanocarriers, thereby enhancing their therapeutic efficacy and safety.

Off-target effects are a significant concern in nanocarrier-based drug delivery. To address this issue, researchers have introduced pH- and ROS-responsive materials for selective drug release in plaques. Additionally, the researchers have modified the nanoparticles by incorporating targeted peptides to increase their uptake by relevant cell types, such as macrophages and foam cells, which are prominent in atherosclerotic plaques. Furthermore, the modification of nanoparticles with mimetic cell membranes (e.g., platelet membranes, macrophage membranes) or high-density lipoproteins has been pursued to promote their targeted accumulation at the plaque site, thereby enhancing the therapeutic specificity and reducing off-target effects.

The stability of nanocarriers in the physiological environment is crucial for effective drug delivery. Researchers have focused on modifying their surface, such as through the application of polyethylene glycol (PEG), which creates a steric barrier, making it challenging for conditioning proteins and other blood components to adhere to liposomes, thereby improving their circulatory stability. Additionally, the introduction of surfactants like poloxamer and sodium dodecyl sulfate has been explored to strengthen nanoparticle stability.

For polymeric micelles, their poor stability in vivo can lead to premature drug leakage. This instability is exacerbated when micelles are diluted in vivo to concentrations below their critical micelle concentration (CMC), causing them to dissociate. Consequently, the selection of micelles with a low CMC is a critical factor in ensuring their stability and efficacy. Researchers have explored the incorporation of additional forces, such as π-π stacking, hydrogen bonding, and host–guest complexation, in conjunction with hydrophobic interactions. These strategies aim to create a more robust and stable micelle structure, thereby improving their performance in drug delivery applications.

Animal models for the investigation of AS need to be reconsidered. Current studies in AS primarily utilize ApoE^−/−^ or Ldlr^−/−^ mouse models. However, these mouse models contain inherent discrepancies with human physiology, such as rarely seen ApoE defects and dissimilar immune systems. Moving forward, the development of animal models with closer similarity to humans is necessary to facilitate more effective clinical translation.

As research progresses, we can anticipate a future where nanotechnology will play a pivotal role in reducing the incidence and severity of atherosclerotic cardiovascular diseases, ultimately leading to better patient outcomes and a healthier population.

## Figures and Tables

**Table 1:** HDL-based NPs developed for atherosclerosis treatment.

Year	Group	HDL	Animal Model	Key Findings	Ref.
2018	Chen	sHDL-T1317	ApoE^−/−^ mice	(1) sHDL itself serving as both a drug carrier and cholesterol acceptor and the LXR agonist (T1317) mediating upregulation of ABC; (2) sHDL minimizing the adverse side effects of T1317 in the liver.	[38]
2019	Liu	LOV-s-rHDL	ApoE^−/−^ mice	(1) LOV-s-rHDL formulated in a 10:1 (LOV:s-rHDL) ratio showed the best synergistic effect; (2) showed a significant reduction in plaque area and MMP levels.	[40]
2020	Dhar	T1T2-MM-HDL-NP	BALB/c mice	(1) Both mitochondria-targeting and MMR-targeting surface functionalities; (2) MRI and therapeutic effects.	[43]
2021	Chen	GM3-rHDL	ApoE^−/−^ mice	(1) pH-responsive release effect; (2) by using GM3-rHDL nanoparticles, low doses of exogenous GM3 can still maintain similar anti-atherosclerotic efficacy.	[44]
2022	Liu	HA-Fc/NP^3^ ST nanoassemblies	Male wild-type C57BL/6 mice	(1) HA targeting; (2) ROS response resulted in smaller carrier size; (3) HA-Fc/NP3 ST resulted in a significant decrease in plaque area, inflammatory factor levels compared to the saline group.	[42]

**Table 2:** Recent research on the use of biomimetic nanocarriers.

Year	Group	Biomimetic NP	Animal Model	Key Findings	Ref.
2019	Wang	RBC/RAP@PLGA	ApoE^−/−^ mice	(1) RBC membranes allow nanoparticles to evade phagocytic system, resulting in long half-life in circulation; (2) good safety.	[71]
2020	Xie	MNC@M-ST/AP	ApoE^−/−^ mice	(1) Leukocyte membrane endows nanoparticles inflammatory tropism.	[72]
2020	Ge	P-Lipo	ApoE^−/−^ mice	(1) Platelet membrane makes nanoparticles inflammatory tropism; (2) mixture of platelet membrane and liposome makes dosage of membrane lower.	[73]
2021	Wang	MM/RAPNPs	ApoE^−/−^ mice	(1) Good biocompatibility and safety; (2) nanoparticles avoid phagocytosis and have targeting.	[74]
2021	Liu	PM-PAAO-UCNPs	partial carotid ligation surgery mouse model	(1) Platelet membrane makes nanoparticles inflammatory tropism; (2) teffect of PDT on atherosclerotic plaque via reactive oxygen species (ROS)-induced apoptosis and regulated lipid metabolism.	[75]
2021	Li	RBC/LFP@PMMP	ApoE^−/−^ mice	(1) RBC membranes allow nanoparticles to evade phagocytic system; (2) drug release in plaque through ROS response bond; (3) integration of diagnosis and treatment.	[56]
2022	Zhang	KPF@MM-NPs	ApoE^−/−^ mice	(1) KPF@MM-NPs treatment significantly reduced the inflammatory response of proliferating macrophages, which was associated with a decrease in key pro-inflammatory cytokines and re-polarization from M1 to M2 phenotype.	[76]
2022	Wang	aCD47@PMSN	ApoE^−/−^ mice	(1) Cell membrane coating prolongs particle circulation by evading immune recognition; (2) significantly reduces atherosclerotic plaque area and stabilizes plaque.	[77]
2022	Yu	PCZ@PB NCs	ApoE^−/−^ mice	(1) Synergistically regulates ROS levels and inflammation, and effectively inhibits foam cell formation; (2) specifically clustered at plaque.	[67]

## Data Availability

No new data were created or analyzed in this study. Data sharing is not applicable to this article.
